# Entomopathogenic fungal infection leads to temporospatial modulation of the mosquito immune system

**DOI:** 10.1371/journal.pntd.0006433

**Published:** 2018-04-23

**Authors:** José L. Ramirez, Christopher A. Dunlap, Ephantus J. Muturi, Ana B. F. Barletta, Alejandro P. Rooney

**Affiliations:** 1 Crop Bioprotection Research Unit, National Center for Agricultural Utilization Research, Agricultural Research Service, United States Department of Agriculture, Peoria, Illinois, United States of America; 2 Laboratory of Malaria and Vector Research, National Institutes of Allergy and Infectious Diseases, National Institutes of Health, Rockville, Maryland, United States of America; Centers for Disease Control and Prevention, Puerto Rico, UNITED STATES

## Abstract

Alternative methods of mosquito control are needed to tackle the rising burden of mosquito-borne diseases while minimizing the use of synthetic insecticides, which are threatened by the rapid increase in insecticide resistance in mosquito populations. Fungal biopesticides show great promise as potential alternatives because of their ecofriendly nature and ability to infect mosquitoes on contact. Here we describe the temporospatial interactions between the mosquito *Aedes aegypti* and several entomopathogenic fungi. Fungal infection assays followed by the molecular assessment of infection-responsive genes revealed an intricate interaction between the mosquito immune system and entomopathogenic fungi. We observed contrasting tissue and time-specific differences in the activation of immune signaling pathways and antimicrobial peptide expression. In addition, these antifungal responses appear to vary according to the fungal entomopathogen used in the infection. Enzyme activity-based assays coupled with gene expression analysis of prophenoloxidase genes revealed a reduction in phenoloxidase (PO) activity in mosquitoes infected with the most virulent fungal strains at 3 and 6d post-fungal infection. Moreover, fungal infection led to an increase in midgut microbiota that appear to be attributed in part to reduced midgut reactive oxygen species (ROS) activity. This indicates that the fungal infection has far reaching effects on other microbes naturally associated with mosquitoes. This study also revealed that despite fungal recognition and immune elicitation by the mosquito, it is unable to successfully eliminate the entomopathogenic fungal infection. Our study provides new insights into this intricate multipartite interaction and contributes to a better understanding of mosquito antifungal immunity.

## Author summary

Fungal biopesticides constitute potential alternative methods of vector control to tackle the rising burden of mosquito-borne diseases and the development of insecticide resistance in mosquitoes. Insect-fungi interactions represent an intricate co-evolutionary arms race between the invading pathogen and its arthropod host. New knowledge gathered through such studies can lead to the design of more effective microbial control strategies. Here we explored the temporospatial interaction of the mosquito *Aedes aegypti* with three different entomopathogenic fungi. Infection assays followed by gene expression studies revealed tissue-specific immune responses that appear to be temporal and fungal strain-specific. Our data shows that fungal infection causes significant reduction in phenoloxidase activity at the later stages of infection. The multifaceted response mounted by the mosquito against the fungal challenge appears to result in the dysregulation of midgut homeostasis, noted by an increase in midgut microbiota, especially in mosquitoes infected with the most virulent strains. Our study demonstrates an intricate mosquito-fungi interaction that, despite fungal recognition and immune response by the mosquito, results in death of the host.

## Introduction

The global increase in new and re-emergent vector-borne diseases has put into perspective once more the need for effective methods of vector control. Although insecticide-based interventions to manage mosquito populations remain important components of vector control programs, the use of biological control agents, such as entomopathogenic fungi, offer promising alternative control methods [1, 2]. In addition to the mosquitocidal properties of entomopathogenic fungi [3–5] fungal infection leads to sub-lethal effects ranging from reduced fecundity, smaller number of gonotrophic cycles [6] and reduced vector competence, all of which are detrimental for vectorial capacity [7, 8]. Host-pathogen interactions represent an intricate co-evolutionary arms race between the invading pathogen and the host. They often encompass an interplay of biological functions, which include immunity, metabolism, and stress management by the host, and the expression of a range of virulence factors by the pathogen, in an effort to overcome the host defenses [9, 10]

In mosquitoes, recent research has uncovered several players involved in fungal recognition, immune elicitation, and anti-fungal defense. For instance, two important components of the antifungal immune response, *CLSP2* and *TEP22*, have been recently identified from infections with the fungi *Beauveria bassiana* [11]. CLSP2 is a specific fungal recognition protein and negative modulator of the host antifungal immunity. *Beauveria bassiana* infection elicits *CLSP2* expression and leads to cleavage of the CLSP2 protein, with its CTL-type domain binding fungal cell components. In turn, Tep22, a thioester-containing protein, functions as anti-fungal effector and its activity is negatively regulated by CLSP2 [11].

Following the infection and pathogen recognition, there is an activation of cellular and humoral immune responses mounted against the invading pathogen [12]. These immune responses are vastly regulated by four innate immune signaling pathways (Toll, IMD, JAK-STAT and JNK) that work in unison to control microbial infections [13–15]. Although the Toll pathway is recognized as the primary innate immune pathway that governs the antifungal response [16], the JAK-STAT pathway has also been implicated in antifungal defense [17]. In comparison, the IMD pathway is better known for playing critical roles in antibacterial and antiplasmodial defenses [18] while the lesser known JNK pathway has been shown to be critical in the antiplasmodial defense and in maintenance of cellular immunity [19, 20].

As entomopathogenic fungi penetrate the insect exoskeleton, they encounter potent cellular and systemic immune responses that include encapsulation, melanization, phagocytosis, and exposure to antimicrobial peptides. Of all these, melanization plays a crucial role in the antifungal systemic immune response [21]. In a tightly regulated reaction, melanization is catalyzed by a series of prophenoloxidase enzymes (PPOs), which leads to the deposition of melanin, and encapsulation of the invading microbe [12]. These anti-fungal immune responses are somewhat effective at initially controlling the fungal infection. Nonetheless, entomopathogenic fungi effectively counteract melanization and other immune defenses by producing metabolites that disrupt anti-microbial activities [22, 23]

In this study, we explored the temporospatial effects of fungal infection in the major arboviral mosquito vector *Ae*. *aegypti*. Infection assays with diverse fungal strains followed by the molecular assessment of infection-responsive genes revealed an intricate interaction between the fungal entomopathogen and the mosquito immune system. Moreover, this study revealed tissue-specific responses that appear to act in concert to limit fungal dissemination. Some of these anti-fungal responses are time and strain-specific. Finally, we show that fungal infection causes significant changes in phenoloxidase activity and ROS which in turn leads to dysregulation of midgut homeostasis. Studies on host responses to microbial pathogens are crucial and should be incorporated in the design of microbial control strategies for a more targeted and effective approach [9]. Although some advancements have been made, most of the studies have focused on the immune responses to either *B*. *bassiana* or *Metarhizium anisopliae* at the early stages of infection. Thus, understanding the interaction between the mosquito vector and entomopathogenic fungi remains an important yet understudied area. Our study assesses the mosquito molecular interactions with four different fungal strains and contributes to a better understanding of mosquito antifungal immunity.

## Materials and methods

### Mosquito rearing

The Rockefeller strain of the mosquito *Aedes aegypti* was reared in standard insectary conditions at 28°C, 70–80% relative humidity, with a 12h light/dark cycle. Adults were maintained on a 10% sucrose solution and blood feeding for egg production was conducted via an artificial membrane feeding system using bovine blood (HemoStat Laboratories Inc.). Larvae were reared on a mixture of rabbit food and tropical fish food. Three to five-day old female mosquitoes were used in all experimental assays.

### Fungal species and exposure assays

Three entomopathogenic strains, *Beauveria bassiana* (MBC 076), *Beauveria brongniartii* (MBC 397) and *Isaria javanica* (MBC 524) were used in the exposure assays (S1 Fig). These were originally isolated from *Anticarsia gemmatalis* (Noctuidae, Lepidoptera); *Melolontha sp*. (Scarabaeidae, Coleoptera) and *Hypothenemus hampei* (Scolytidae, Coleoptera), respectively. The assays also included exposure to a non-entomopathogenic fungi, *Trichothecium roseum* (MBC 071), a saprophyte originally isolated from a syrphid fly (Syrphidae, Diptera). Fungal cultures were grown on Sabouraud dextrose agar and yeast extract (SDAY) medium and incubated at 26°C for 15 days. Conidial oil suspensions were prepared by scrapping the sporulating surface with soy bean oil, creating a homogeneous suspension via lightly mixing using an electronic pestle to break up aggregates, and then filtering the suspension through a cheese cloth to remove mycelia. Soy bean oil has been shown to be an effective carrier in oil-based formulations [24, 25], maintaining conidial viability while allowing impregnation to the hydrophobic mosquito cuticle. Conidial concentrations were determined by direct counts using an improved Neubauer hemocytometer and adjusted to a final concentration of 1x 10^8^ conidia/mL. For mosquito exposure assays, 3–5 day old mosquitoes were cold-anesthetized, and 36.8 nl of the adjusted conidial oil suspension was topically applied to the ventral surface of the coxal region of mosquitoes using a Nanoject II micropipet. The small volume and location of the application site was chosen following tests and to avoid occlusion of the mesothoracic spiracles. A control group was exposed to the same volume of soy bean oil devoid of fungal spores. To assess the effect of bacteria on mortality of fungal-infected mosquitoes, insects were maintained on either a 10% sucrose solution containing penicillin (20 units/mL) and streptomycin (20 μg/mL) or in 10% sucrose solution alone (controls). Fresh antibiotic solution was provided daily from the day prior to the fungal infection until the end of the experiment. To assess whether any bacterial-effects on survival would be accentuated by a stronger fungal challenge, antibiotic-treated and untreated controls were exposed to a higher dose of fungal conidia (50.6 nl of a 1x 10^9^ conidia/mL suspension). Fresh conidial suspensions from new batches of conidia were prepared for each experimental exposure assay, and a new batch of mosquitoes were used in each experiment. At least three independent experiments were conducted for each procedure involving fungal infection. Following fungal challenge, mosquitoes were maintained under standard insectary conditions with *ad libitum* access to 10% sucrose solution. Mortality was evaluated daily, and mosquito cadavers were removed from the cages and checked for fungal growth via maintaining them in sealed petri dishes containing moist filter paper. The survival curves were compared using Kaplan-Meier with Log-rank test to evaluate significance (GraphPad 7.0). LT_50_ and LT_95_ values were calculated by probit analysis using SAS 9.4 statistical package.

### Fungal identification

For fungal identification, DNA was extracted using CTAB extraction buffer (Sigma) while polymerase chain reaction (PCR) amplification of the partial Translation elongation factor (TEF) gene was conducted using 1x AmpliTaq Gold 360 Master Mix (Applied Biosystems), ~100 ng template DNA, and 0.2 μM each of primers 983f and 1567r [26]. The TEF gene PCR reactions were performed with an initial denaturation step of 10 min at 95°C followed by 35 cycles, each consisting of a 30 s denaturation step at 95°C, a 30 s annealing step at 55°C, and a 1 min extension step at 72°C, and then followed by a final extension step for 7 min at 72°C. Amplification products were purified using Montage PCR Cleanup Filter Plates (Millipore). Sequencing reactions were conducted using the ABI BigDye version 3.0 sequencing kit (Applied Biosystems, Foster City, CA) following the manufacturer’s suggested protocol, but at one-tenth the recommended volume. Reaction products were purified using the BigDye XTerminator Purification Kit (Applied Biosystems) following the manufacturer’s suggested protocol and sequenced on an ABI3730 genetic analyzer (Applied Biosystems) using the aforementioned oligonucleotide primers. Resulting DNA sequences were visually edited and assembled using Sequencer 5.4 software (Gene Codes Corporation). Consensus sequences were aligned using CLCBio genomics workbench 9.0 software (Qiagen Inc.). Phylogenetic analysis was performed in MEGA 7 [27] using the Maximum Likelihood method with a General Time Reversible model. The complete deletion option was used, and the level of bootstrap support was calculated from 1000 replicates (S1 Fig).

### Conidia, hemocyte and blastospores staining

Conidial spores were collected from two-week-old cultures and stained with lactophenol cotton blue stain (Invitrogen) and imaged using a 40x objective on a Zeiss Axioplan compound microscope. For the imaging of hemocytes and blastospores, infected mosquitoes were cold-anesthetized and their hemolymph perfused with 1x PBS at 6d PI. The drop of hemolymph was collected on a microscope slide, air dried, fixed with cold methanol and stained with Giemsa stain (Invitrogen). The slides were rinsed with water before observation using a 40x objective on a Zeiss Axioplan compound microscope. Images were captured using the Leica DMC2900 microscope camera (Leica)

### Gene expression

For gene expression, midgut and abdominal fat body walls from fungal-infected mosquitoes were dissected on a drop of 1x PBS at 3 and 6d PI. The abdominal fat body walls, comprised primarily of trophocytes and oenocytes (ectodermic cells), were devoid of Malpighian tubules, crop or ovaries but are expected to include associated tracheoles, sessile hemocytes, peripheral neurons and pericardial cells [28–30]. Then, midguts and fat body walls were homogenized in TRIzol (Invitrogen) and processed for RNA according to the manufacturer’s instructions. RNA concentration and quality were assessed via NanoDrop (Thermo Scientific), and cDNA synthesis was conducted on normalized amounts of RNA using the QuantiTect reverse transcription kit with DNA Wipeout (Qiagen). Two microliters of the generated cDNA were used in a 10 μl qPCR reaction using the PowerUp SYBR green Master mix qPCR kit (Qiagen) with gene specific primers (S1 Table). The qPCR cycling conditions were those recommended for the master mix and consisted of holding at 95.0°C for 10 min and 40 cycles of 15 s at 95.0°C and 1 minute at 60°C. A melt curve stage at the end of the reaction was included. For each experiment, the relative quantification of transcript abundance was done in two groups of midguts or their corresponding fat body walls dissected from 10 mosquitoes. Each sample was analyzed in duplicates (technical replicates) and the reproducibility of the results were evaluated via three independent experiments conducted on separate dates with different batches of mosquitoes and using fresh conidial suspensions for each infection. The ribosomal protein Rp49 gene was used for normalization of cDNA templates. This gene has been successfully used in expression profiles involving *Ae*. *aegypti* [31–33]; including transcriptomic studies on mosquito-fungal interactions [4, 34]. The qPCR assays were ran on Applied Biosystems 5700 Fast Real-time PCR (Applied Biosystems) and the data was analyzed post run using the ΔΔCt method [35]. The statistical significance of fold change values was determined on log_2_ transformed values via ANOVA with Dunnett’s post-test in Prism (GraphPad). Heat maps were created using Morpheus (Broad Institute, https://software.broadinstitute.org/morpheus/) from the median log_2_-fold change values of at least 3 independent experiments.

### Reactive oxygen species (ROS) levels

The Amplex Red reagent (Invitrogen) was used to determine the levels of hydrogen peroxide release (ROS) in the midguts of fungal-infected mosquitoes following the manufacturer’s recommendations. In short, midguts were dissected in PBS under a chill-block at 4°C and pooled into five midguts per group for evaluation of H_2_O_2_ levels. Samples were incubated at room temperature for 30 min with 100 μM Amplex Red reagent and 2 units of horseradish peroxidase (HRP). Samples were then spun, the supernatant collected, and fluorescence measured with Molecular Devices M5 (Ex: 530 nm; EM: 590 nm) along with a hydrogen peroxide standard curve. A non H_2_O_2_ blank sample was also included in the readings.

Mosquito midguts were also stained with dihydroethidium (DHE) (Invitrogen) to assess superoxide levels [36] following the protocol from Xiao et al [37]. In short, mosquito midguts were dissected at 6d PI in a solution of PBS and 2 mg/mL of 3-amino-1,2,4-triazole (Sigma). Dissected midguts were then incubated with 2 μM DHE in 1x PBS at room temperature for 30 min in the dark, washed twice with 1x PBS and then fixed with 4% paraformaldehyde for 30 minutes. Midguts were then washed twice before being placed on a slide. Nuclei was stained with DAPI (Invitrogen) and the slides were imaged using a 4x objective lens on an EVOS FL Auto (Life Technologies) fluorescent microscope. Images were acquired using the same fluorescent levels and under identical conditions (exposure time, microscope, reagents, etc.) for all tested groups.

### Phenoloxidase (PO) activity

To assay the level of PO activity in mosquitoes, we followed the protocol from Sadd et al. [38] with modifications. In short, mosquitoes were collected at 3 and 6d PI, and two whole mosquitoes were pooled per sample (10 samples per treatment) and homogenized for 30 s with a 2.4 mm bead and 50 μl of 1x PBS using the TissueLyser II (Qiagen). Homogenates were then centrifuged (3000 rpm, 4°C, 5 min), and then 35 μl of the supernatant was snap frozen in liquid nitrogen and stored at -80°C for subsequent analyses. The enzymatic reaction assay was conducted by thawing the samples on ice and adding 15 μl of the sample or 15 μl of PBS (negative control) to a flat-bottomed 96-well plate containing 20 μl PBS and 140 μl of molecular-grade water. Subsequently, 20 μl of a solution of molecular grade water mixed with L-Dopa (4 mg per mL H_2_O; 3,4 dihyroxy-L-phenylalanine) was added to each well. Plates were shaken for 5s at 30°C in a spectrophotometer (Multiskan GO, Thermal Scientific), with absorbance read at 490 nm every 15 s with 5 s shaking between reads. PO activity was measured from the slope (V_max_) of the reaction curve in its linear phase over 160 readings. Samples were read in duplicate and the average was used in further analyses. Ten samples per treatment were used in each experiment with three independent experiments conducted.

### Bacterial load determination and bacterial acquisition post-antibiotic treatment

Bacterial load was determined via qPCR on both the cDNA of mosquito midguts used for gene expression analyses and from genomic DNA extracted from the midguts of separate cohorts of fungal-infected mosquitoes. DNA was extracted from pools of 10 midguts using the DNeasy blood and tissue kit (Qiagen) kit according to the manufacturer’s instructions for bacterial DNA extraction. Amplification was conducted using *16s* ribosomal RNA universal primers (S1 Table) following the same conditions as for gene expression assays stated above. The *Ae*. *aegypti* ribosomal protein *Rp49* and the ribosomal protein *Rps17* gene were used for normalization of cDNA and DNA templates respectively. To normalize the amount of bacteria present in our cohort of mosquitoes before fungal infection, mosquitoes were treated with antibiotics (20 μg/mL streptomycin and 20 units/mL penicillin) *ad libitum* for two days, maintained in sterile sucrose for one more day and then fed on a cocktail of bacteria via sugar meal for 24 h prior to fungal challenge. Bacterial isolates used in this study were the most commonly isolated species from our lab-reared mosquitoes and identified as: *Serratia marcescens*, *Burkholderia spp*., *Leclercia spp*., and two species of *Pseudomonas spp*. Bacteria were grown overnight, spun at 3,000 rpm for 5 min and washed twice in 1x PBS. They were then adjusted to 1x 10^4^ for each bacterium and mixed in a 3% sucrose solution prior to their use. Before dissection, mosquitoes were surface-sterilized with 3% bleach for 2 minutes, 70% EtOH for 2 minutes and washed twice with 1x PBS. Midguts were dissected on a drop of sterile 1x PBS and then pooled in groups of 10 midguts per sample. Midguts were homogenized using the TissueLyser II (Qiagen) and DNA was extracted with the DNeasy Blood and Tissue kit (Qiagen). Their concentration and quality were analyzed via NanoDrop (Thermo Scientific), and employed in subsequent Illumina sequencing.

### Microbiome analysis of fungal-infected and uninfected controls

The midgut microbiome of control and fungi-challenged mosquitoes following the reintroduction of bacteria post-antibiotic treatment was analyzed via 16s rRNA sequencing. Ten midguts were pooled for each treatment with the analysis conducted in four to five replicates. PCR was performed using Kapa HiFi PCR mastermix (Kapa Biosystems Willington, MA) using the following parameters; 95°C, 10 min, 35 cycles of 95°C, 30 s; 58°C, 30 s; 72°C, 60 s. PCR primers for the bacterial community (341f and 806r) targeted the V3-V4 region of the 16S rRNA genes [39]. The loci-specific primers were incorporated into fusion primers for Illumina dual indexing and incorporation of Illumina adapters [40]. After sequencing, the amplicons were cleaned and normalized using a SequalPrep normalization plate (Thermo Fisher Scientific, Waltham, MA). The samples were pooled and the library quantified with a Kapa library quantification kit (Kapa Biosystems Willington, MA). The samples were sequenced using an Illumina MiSeq system with a MiSeq V3 2 x 300 bp sequencing kit. The demultiplexed reads were quality trimmed to Q30 using the CLC genomics workbench v9.5 (Qiagen inc., Valencia, CA). Read pairing, fixed length trimming and OTU clustering was conducted using the CLC Bio Microbial Genomics module (Qiagen inc., Valencia, CA) using the reference sequences from the Greengenes ribosomal ribonucleic acid gene database (97% similarity) [41]. OTUs with five or more sequence reads were used in subsequent analyses. Alpha diversity was evaluated using Chao1, Shannon, and Simpson indices; while the beta diversity was analyzed by using the principal coordinate analyses (PCoA) based on the Bray-Curtis dissimilarity matrix. The diversity indices were tested for normality via the Shapiro-Wilk normality test, those passing the normality test were analyzed via ANOVA with the Dunnett’s multiple comparison test. Otherwise, they were analyzed via the Kruskal-Wallis test for multiple comparison. Diversity measurements were performed on PAST 3.15 software and the statistical analysis was conducted with GraphPad Prism 7 (GraphPad).

### Statistical analysis

All statistical analyses to assess significance of Kaplan-Meier survival curves, gene expression analyses, enzymatic activity, and biodiversity index comparisons were performed using GraphPad Prism 7 (GraphPad). Significance was assessed at *P*<0.05 with asterisks indicating the strength of the significance (*P< 0.05; **P<0.01; ***P<0.001). Error bars represent the standard error of the mean and the type of test used is indicated in the respective figure legend.

## Results

### Mosquito survival post-exposure to fungal conidia

**Fig 1:**
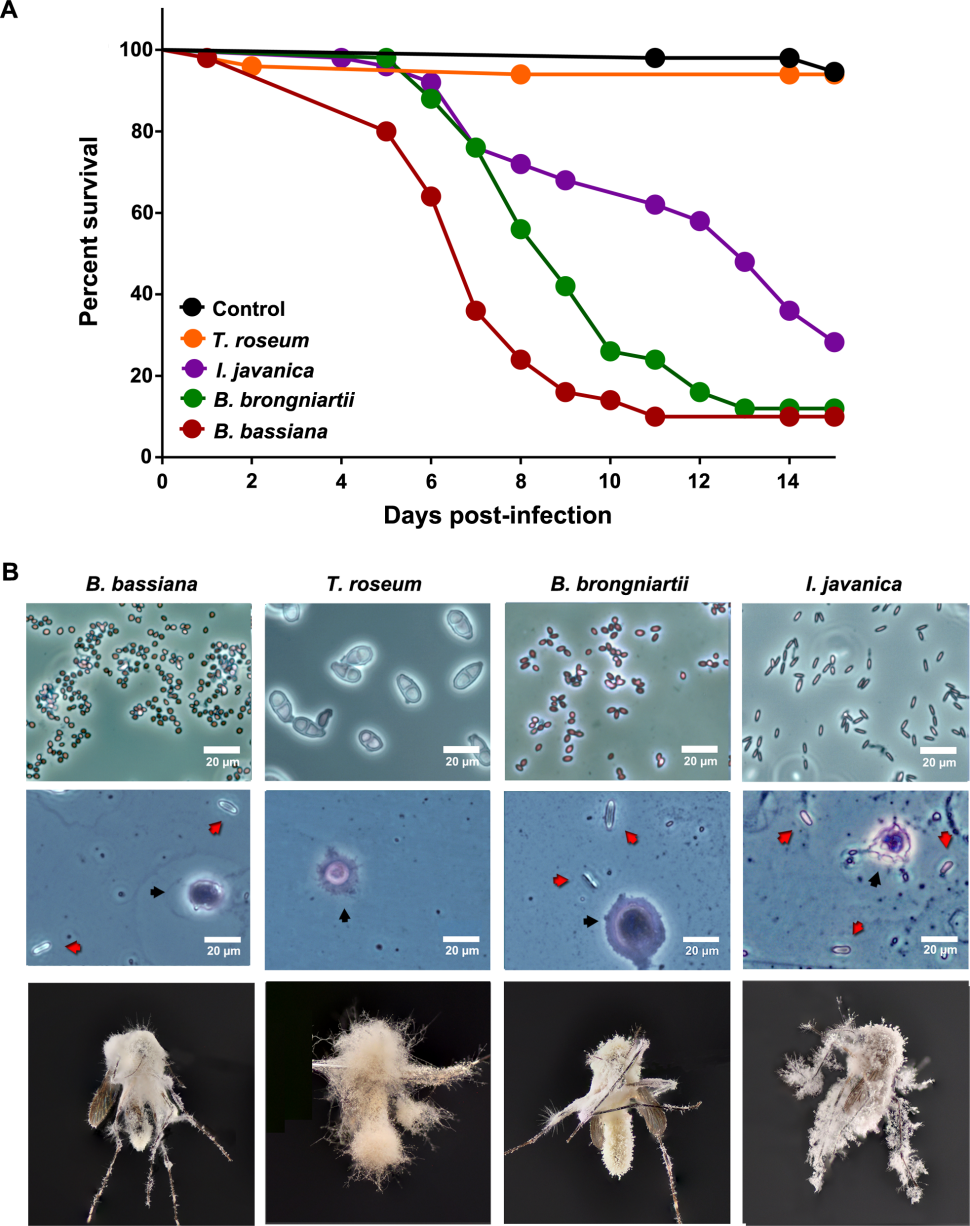
Mosquito infection by diverse fungal strains. **(A)** Survival curves of fungal-challenged mosquitoes. Graph represents 3 independent experiments and data was analyzed with Log-rank Test (GraphPad Prism 7) **(B)** Entomopathogenic fungi infection stages: conidia (top), hemocytes and blastospores (middle), and hyphal growth on mosquito cadavers (bottom). Black arrows indicate hemocytes while red arrows indicate blastospores. No blastospores were found in *T*. *roseum*-challenged mosquitoes.

Mosquito survival differed significantly following challenge with the four fungal species (log-rank Mantel–Cox test, *X*^2^: 181.4, *P*<0.0001) (Fig 1A). Mosquitoes topically infected with *B*. *bassiana* had the highest mortality (94.9%, *X*^2^: 86.33, *P*<0.0001). The second and third most virulent species were *B*. *brongniartii* with 88% (*X*^2^: 81.46, *P*<0.0001) and *I*. *javanica* with 71.8% mortality (*X*^2^: 47.66, *P*<0.0001). The low mortality (6%) observed in mosquitoes challenged with the saprophytic isolate *T*. *roseum* was not significantly different from the control group (4%, *X*^2^: 0.2409, *P* = 0.6236). The values for LT_50_/LT_95_ were determined for each pathogenic strain and varied with 7.39 days for *B*. *bassiana*, 9.25 days for *B*. *brongniartii* and 12.44 days for *I*. *javanica* (S2 Table). LT_50_ could not be estimated for *T*. *roseum* due to mosquito survival exceeding 50% or 95% throughout the experiment. Slide preparations from fungal spores confirmed both conidial morphology and purity of the samples (Fig 1B, top panel). Fungal bodies were present in the hemolymph of mosquitoes challenged with *B*. *bassiana*, *B*. *brongniartii* and *I*. *javanica* (Fig 1B, middle panel) at 6d PI, confirming the success of fungal infection. We did not observe any blastospores or fungal bodies in the hemolymph preparations of the *T*. *roseum*-challenged mosquitoes. Cadavers collected soon after death showed mycelial growth and sporulation for all of the fungal-challenged groups (Fig 1B, lower panel), but those were absent in the few control group cadavers.

### Fungal recognition in fat body and midgut tissues

**Fig 2:**
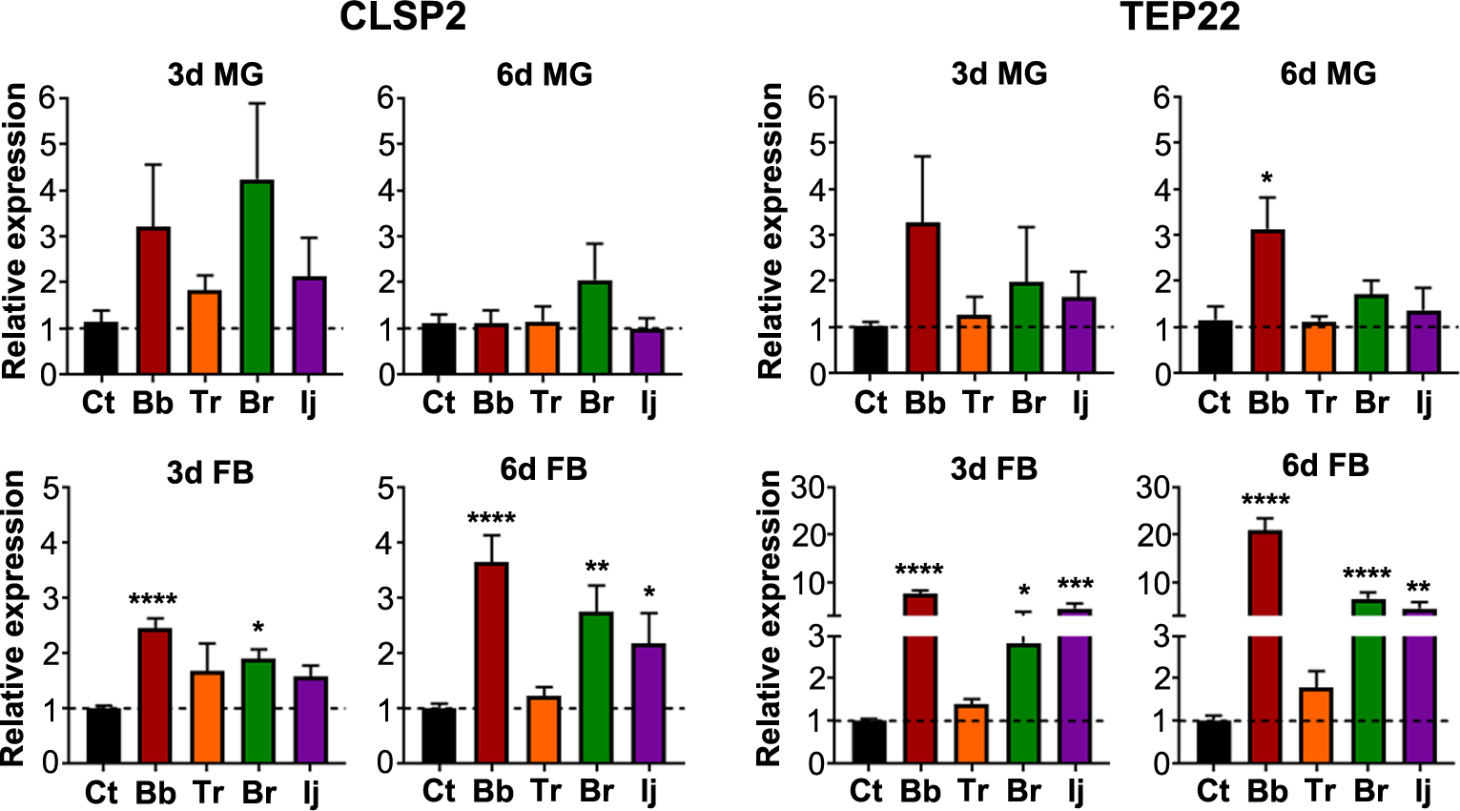
Recognition of diverse fungal strains by the mosquito immune system. Relative expression of fungal recognition genes *CLSP2* and *TEP22* in the midgut and fat body at 3d and 6d PI. Data represents the fold change in expression from three independent experiments. Data was analyzed by one-way ANOVA with Dunnett’s post-test; * *P*<0.05, ** *P*<0.01, *** *P*<0.001, **** *P*<0.0001.

To assess successful fungal infection and recognition by the mosquito immune system, we evaluated the expression of two genes, *CLSP2* and *TEP22*, which have been found to be significantly expressed in response to *B*. *bassiana* infection at 24h post-exposure [11]. *CLSP2* is composed of a serine protease and a C-terminal galactose-type C-type lectin domain and acts as a negative regulator of the antifungal immune response [11]. *CLSP2* is upregulated at 3d PI in the fat body of mosquitoes infected with *B*. *bassiana* and *B*. *brongniartii*, and shows a slight increase, albeit not significant, in mosquitoes challenged with *I*. *javanica* or *T*. *roseum* (Fig 2). This increase in fat body *CLSP2* expression became more prominent at 6d PI for all fungi-infected mosquito groups except for *T*. *roseum*-challenged mosquitoes (ANOVA, with Dunnett’s test, *P* = 0.874).

Although, *CLSP2* and *TEP22* have been observed to primarily be expressed in the fat body, our expression analysis shows an upward trend at 3d PI in the midgut of all infected mosquitoes (Fig 2). While not significant, by 6d PI it was no longer observed in infected mosquitoes; with all *CLSP2* upregulation occurring exclusively in the fat body. *TEP22* regulation in the midgut was observed at 3d and 6d PI, but overall it was significantly upregulated only in the midgut of mosquitoes infected with *B*. *bassiana*. In comparison, *a* significant increase in fat body *TEP22* expression was observed at 3d and 6d PI in mosquitoes infected with *B*. *bassiana*, *B*. *brongniartii* and *I*. *javanica* (Fig 2). The fat body of *T*. *roseum*-challenged mosquitoes showed no significant increase in *TEP22* expression.

### Fungal infection leads to tissue-specific and time-dependent elicitation of immune pathways and antimicrobial effectors

**Fig 3:**
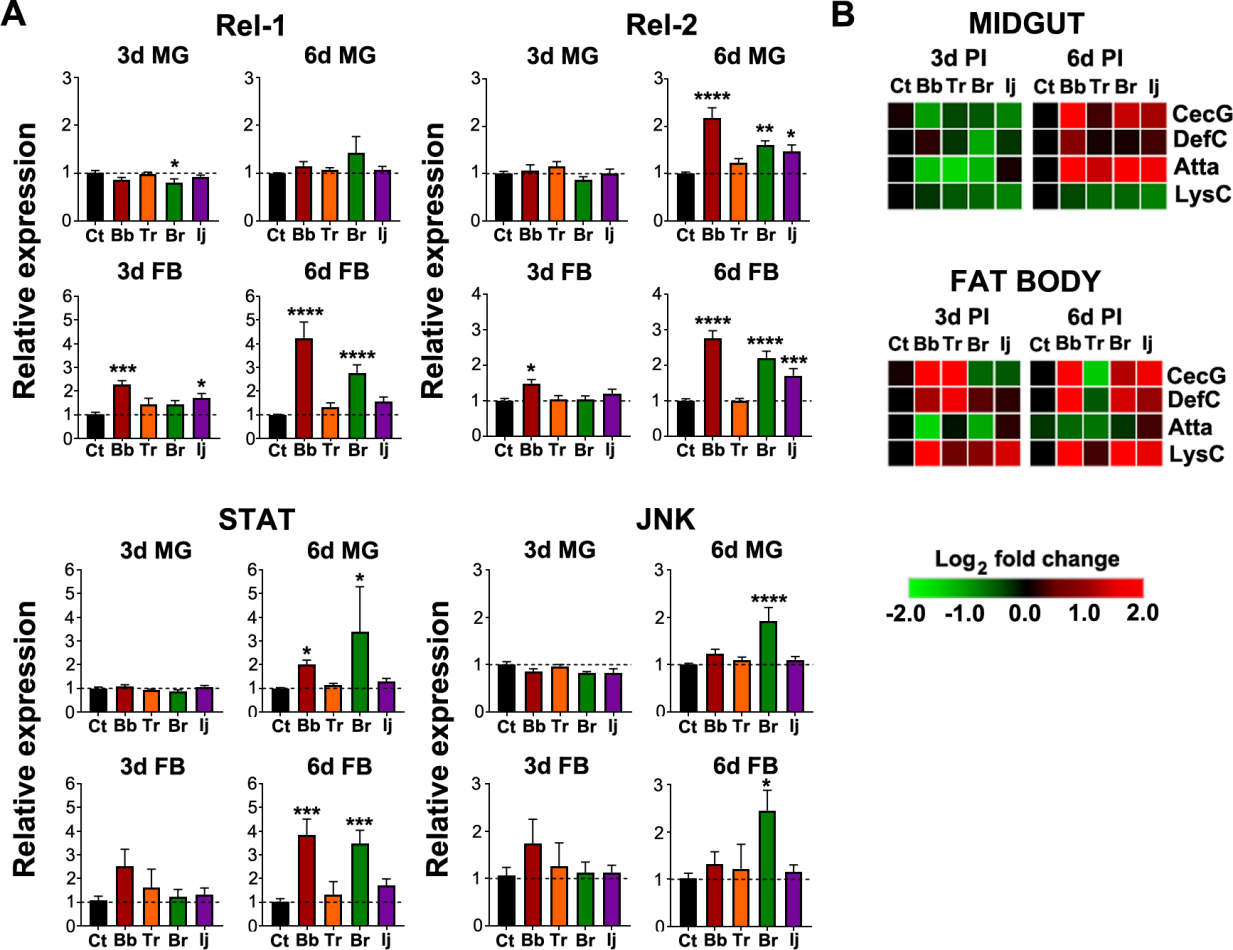
Elicitation of innate immune signaling pathways following fungal infection is time, tissue and fungal strain-specific. **(A)** Gene expression analysis of *REL1* (Toll pathway), *REL2* (IMD pathway), *STAT* (JAK-STAT pathway) and *JNK* (JNK pathway) in the midgut and fat body at 3d and 6d PI. Data represents the fold change in expression from at least three independent experiments. **(B)** Heatmap generated from the antimicrobial peptide gene expression following fungal infection in the midgut and fat body at 3d and 6d PI. Heat-map from qPCR data represents the median of log_2_ fold change values from three independent experiments with red representing higher expression levels and green lower expression levels compared to the control. *CECG*, cecropin G; *DEFC*, defensin C; *ATTA*, attacin; *LYSC*, lysozyme C. Data was analyzed by one-way ANOVA with Dunnett’s post-test; * *P*<0.05, ** *P*<0.01, *** *P*<0.001, **** *P*<0.0001.

To assess fungal elicitation of the innate immune pathways, we evaluated the expression of the transcription factors *REL1*, *REL2*, *STAT*, and pathway component *JNK*, in the midgut and fat body at 3 and 6 days PI. These pathway components belong to the four-main mosquito immune pathways Toll, IMD, JAK-STAT and JNK respectively. *REL1* expression was significantly downregulated in the midgut of *B*. *brongniartii*-infected mosquitoes at 3d PI but returned to the control levels at 6d PI (Fig 3A). No other modulation of this transcription factor was observed in the midgut at 3d or 6d PI with the other fungal strains. In contrast, *REL1* expression was significantly upregulated in the fat body at 3d PI in *B*. *bassiana* and *I*. *javanica*-infected mosquitoes with stronger expression in the fat body of mosquitoes infected with *B*. *bassiana* and *B*. *brongniartii* at 6d PI. No significant *REL1* regulation was observed in *T*. *roseum*-infected mosquitoes (Fig 3A). In comparison to *REL1* (Toll pathway), the IMD pathway transcription factor *REL2* had no modulation of expression at 3d PI but was strongly upregulated in the midguts of *B*. *bassiana*, *B*. *brongniartii* and *I*. *javanica*-infected mosquitoes at 6d PI. No significant modulation of *REL2* expression was observed in the midgut of *T*. *roseum*-infected mosquitoes.

As for the fat body tissues, *REL2* expression at 3d was only slightly upregulated in mosquitoes infected with *B*. *bassiana* but had a robust upregulation at 6d PI in mosquitoes infected with either of the two *Beauveria* species or *I*. *javanica*, but with no change in the *T*. *roseum*-challenged group. *STAT* showed no significant regulation in the midgut at 3d PI and a slight upregulation at 6d PI in *B*. *bassiana* and *B*. *brongniartii*-infected mosquitoes. The fat body showed similar trends, with no significant fat body *STAT* expression at 3d but with a significant increase only in *B*. *bassiana* and *B*. *brongniartii*-infected mosquitoes at 6d PI. *JNK* expression was not significantly modulated at 3d PI in the midgut or fat body but presented a strong significant upregulation at 6d PI only in the midgut and fat body of *B*. *brongniartii-*infected mosquitoes (Fig 3A).

We then evaluated the expression of four antimicrobial peptides, cecropin G (*CECG*), defensin C (*DEFC*), attacin (*ATTA*) and lysozyme C (*LYSC*). We observed a dynamic modulation of expression that was also tissue-specific, time and fungal-isolate dependent (Fig 3B, S2 Fig). *Cecropin G*, *DEFC* and *ATTA* showed no significant changes in expression in midguts or fat body tissues at 3d PI but a strong *CECG* upregulation by 6d PI only in *B*. *bassiana-*infected mosquitoes. The expression of attacin in midgut and fat body tissues presented an irregular modulation across the biological replicates; and as a group it was not statistically significant. We also observed weak modulation of *DEFC* with a significant upregulation at 6d PI only in the fat body of *B*. *bassiana*-infected mosquitoes. Lysozyme C was the only effector that consistently show downregulation in the midgut and upregulation in the fat body of fungal-infected mosquitoes. In particular, *LYSC* was significantly downregulated in the midguts of *B*. *brongniartii* and *I*. *javanica*-infected mosquitoes at 6d PI. In contrast, *LYSC* expression was significantly upregulated at 3d and 6d PI in the fat body of mosquitoes infected with the entomopathogenic strains *B*. *bassiana*, *B*. *brongniartii* and *I*. *javanica*. No significant *LYSC* modulation was observed in *T*. *roseum*-challenged mosquitoes (Fig 3B and S2 Fig).

### Fungal infection leads to downregulation of PPO gene expression and PO activity

**Fig 4:**
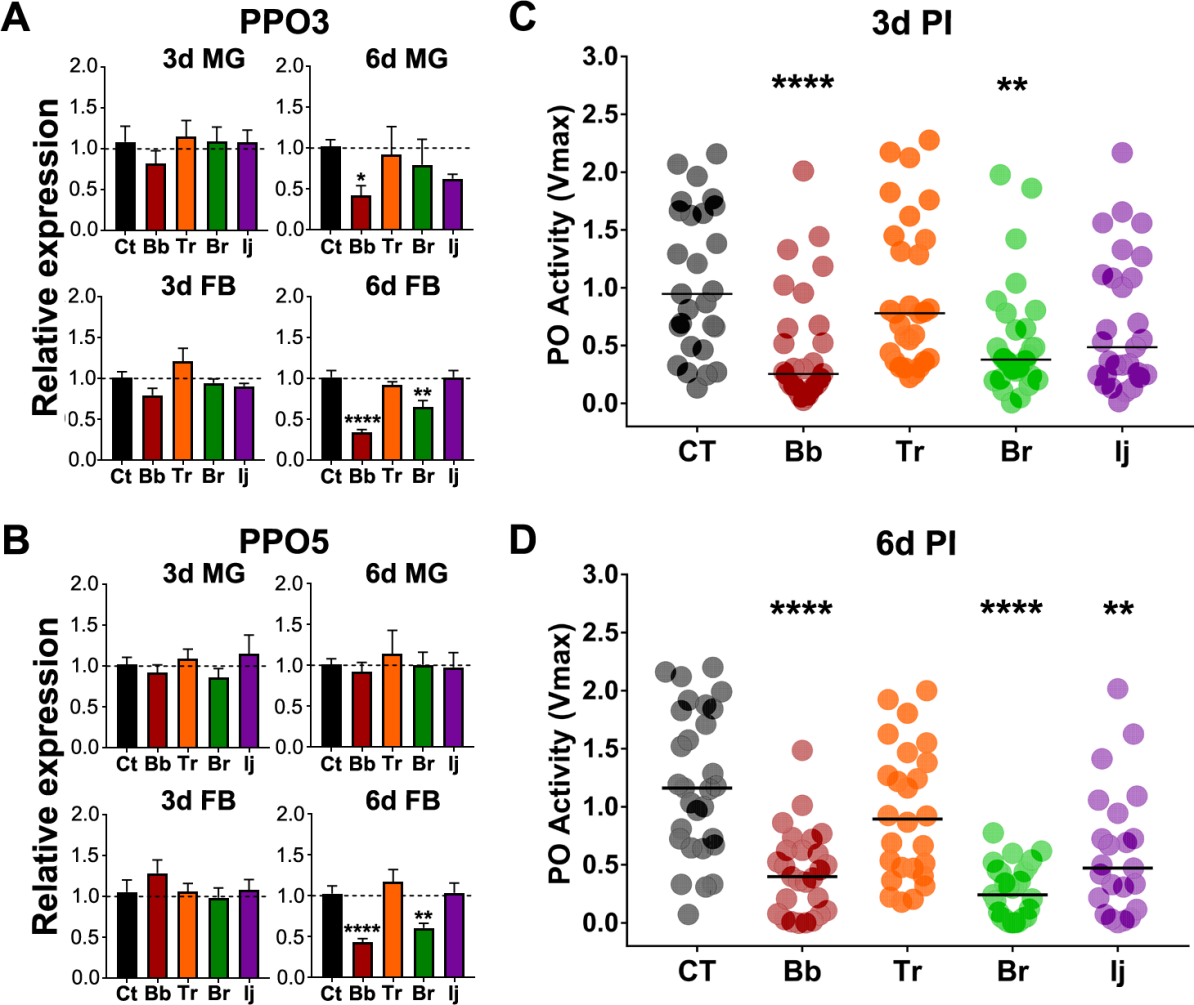
Entomopathogenic fungal infection results in the downregulation of PPO gene expression and reduction of phenoloxidase activity. Gene expression profiles of **(A)** *PPO3* and **(B)** *PPO5* in the midgut and fat body at 3d and 6d PI. Data represents the fold change in expression from three independent experiments. Data was analyzed by one-way ANOVA with Dunnett’s post-test. * *P*<0.05, ** *P*<0.01, *** *P*<0.001, **** *P*<0.0001. Phenoloxidase activity (Vmax) was evaluated from whole-body macerates of mosquitoes at **(C)** 3d and **(D)** 6d PI. Data represents samples from 3 independent experiments. Data was analyzed via ANOVA-Kruskal-Wallis followed by Dunn’s multiple comparison test. * *P*<0.05, ** *P*<0.01, *** *P*<0.001, **** *P*<0.0001.

We investigated the expression of two important gene members of the phenoloxidase cascade (*PPO3* and *PPO5*). We observed a decrease in *PPO3* and *PPO5* expression in the midgut and fat body of *B*. *bassiana* and *B*. *brongniartii*-infected mosquitoes at 3d PI with a more pronounced downregulation in fat body tissues at 6d PI (Fig 4A). The *PPO3* and *PPO5* expression levels in mosquitoes infected with *T*. *roseum* and *I*. *javanica* was not-significant relative to the control (Fig 4A and 4B). We next measured the whole body phenoloxidase (PO) enzymatic activity at 3d and 6d PI. We observed a significant decline in PO activity in mosquitoes infected with *B*. *bassiana* and *B*. *brongniartii*, and non-significant decline in *I*. *javanica*-infected mosquitoes at 3d PI (Fig 4C). This reduction in PO activity was stronger at 6d PI in all mosquitoes exposed to either *B*. *bassiana*, *B*. *brongniartii* or *I*. *javanica* (Fig 4D). No change was observed in mosquitoes challenged with the saprophytic isolate *T*. *roseum* (Fig 4C and 4D).

### Midgut bacterial load increases as a result of fungal infection

**Fig 5:**
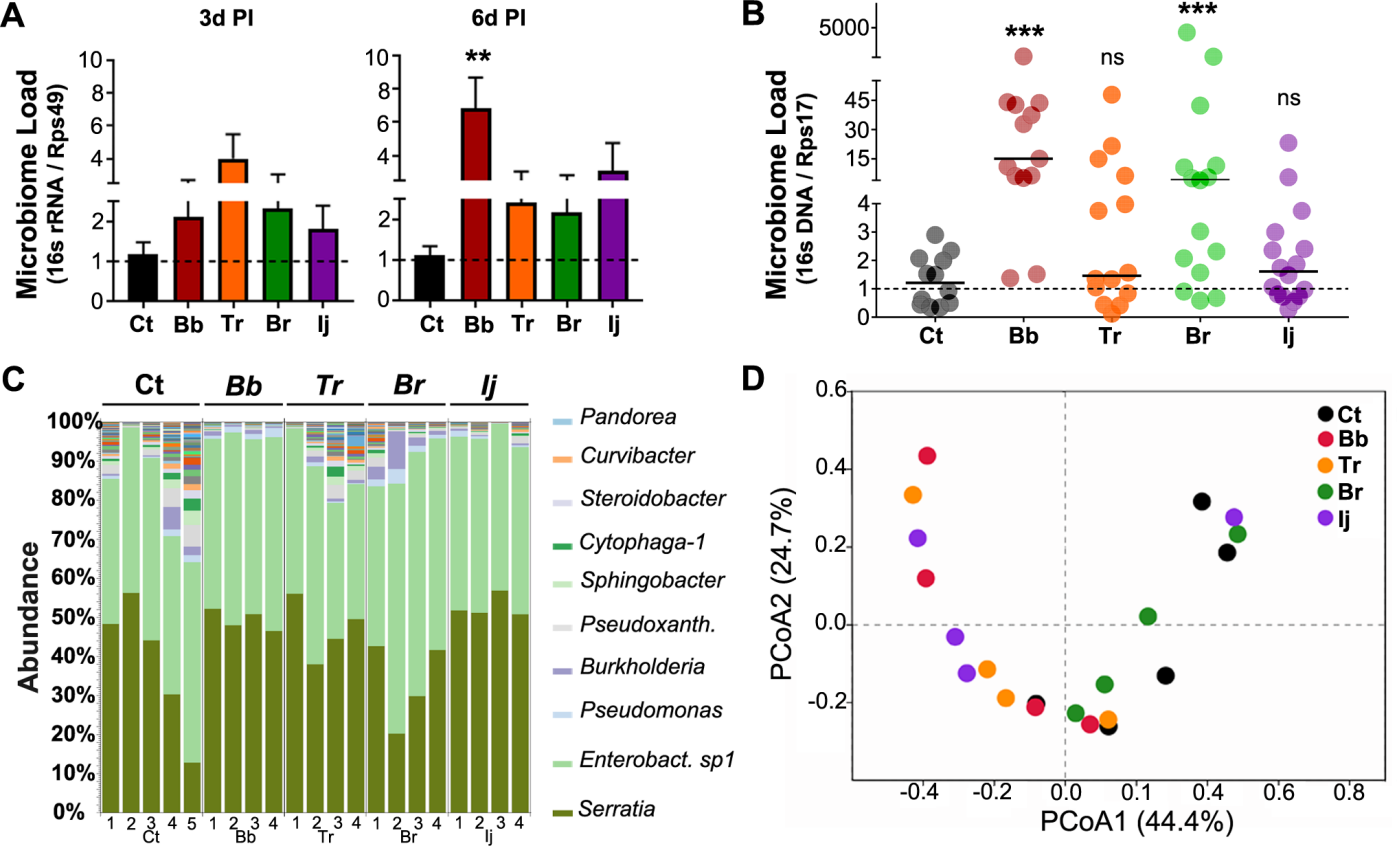
Entomopathogenic fungal infection leads to dysregulation of the mosquito midgut homeostasis. Relative quantification of **(A)** bacterial 16s rRNA in the mosquito midgut at 3d and 6d PI and **(B)** bacterial 16s rDNA at 6d PI. ** *P*<0.01. **(C)** Relative bacterial OTU abundance at the genus level from mosquito midguts collected at 6d PI. Labels on the x-axis represent the replicate groups under each treatment. **(D)** Principal coordinate analysis (PCoA) depicting patterns of beta diversity for the mosquito midgut bacterial communities under different fungal infections. PCoA was based on the Bray-Curtis dissimilarity matrix.

We decided to evaluate whether the modulation of the mosquito immune system by the fungal challenge could be reflected in the midgut homeostasis and affect microbial load. Measuring the bacterial 16s rRNA relative to mosquito Ribosomal protein 49 (Rp49), at 3d and 6d PI, showed an upward trend in the midgut bacterial load of mosquitoes infected with *B*. *bassiana* and *B*. *brongniartii*, with less of an increase in mosquitoes infected with *I*. *javanica* (Fig 5A). This increase, was significant in the midgut of *B*. *bassiana*-infected mosquitoes at 6d PI. To corroborate our transcript results, we evaluated the levels of 16s rDNA via qPCR. The results were in accord to our transcript analysis with significant increase in bacterial 16s rDNA copies in mosquitoes infected with *B*. *bassiana* and *B*. *brongniartii* (Fig 5B).

We next evaluated whether these changes were due to an overall increase in the midgut bacterial load or to a specific bacterial taxon, one benefiting from the midgut physiological conditions created in response to the fungal-challenge. Our microbiome study indicated no significant difference in the midgut microbial composition among all treatments. Although there was a slight increase in microbial diversity in control mosquitoes at the family and genera level (Fig 5C and S3A Fig), the OTU calculated species richness (Chao1), Shannon’s diversity index and Simpson’s diversity index did not change significantly from the controls (*P* > 0,05) (S3B Fig) The microbial community was dominated by two Enterobacter species with *Serratia marcescens* being the most abundant of all OTUs across all sample treatments. Other represented genera included *Pseudomonas spp*., *Burkholderia spp*., *Pseudoxanthomonads spp*., and *Xanthomonads spp*. (Fig 5C). For beta diversity analysis, we evaluated the relationship between entomopathogenic fungal infection and mosquito midgut microbiota using the principal coordinate analysis (PCoA) based on the Bray-Curtis dissimilarity matrix. The PCoA test showed that the community composition did not differ among treatments (Fig 5D).

### Fungal infection-responsive modulation of immune genes and reduction in ROS activity leads to increase in midgut bacteria

**Fig 6:**
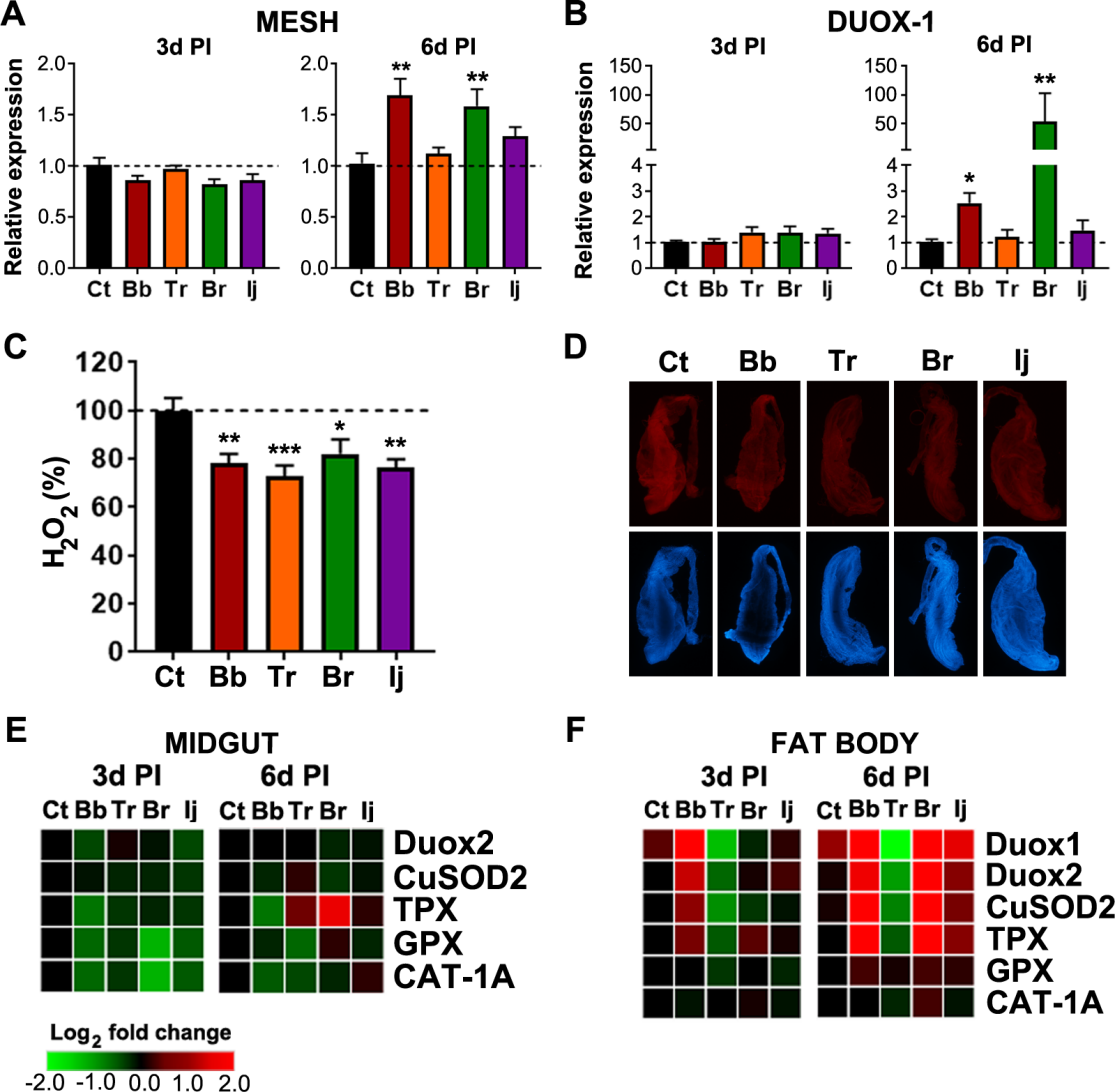
Dysbiosis of the mosquito gut occurs via modulation of gut-homoeostasis-related genes and reduction of ROS activity in the midgut. Expression profiles of **(A)** *MESH* and **(B)** *DUOX1* in the mosquito midgut at 3d and 6d PI. Data represents the fold change in expression from at least three independent experiments. **(C)** Hydrogen peroxide release (ROS) from the mosquito midgut at 6d PI. **(D)** Midgut ROS activity was imaged using DHE at 6d PI. Top panels show DHE fluorescence (red) while lower panels show DAPI staining of nuclei (blue). Heat map depicting the differential expression of oxidant and antioxidant genes in the **(E)** midgut and **(F)** fat body at 3d and 6d PI. Data represents the fold change in expression from three independent experiments. Data was analyzed by one-way ANOVA with Dunnett’s post-test. * *P*<0.05, ** *P*<0.01, *** *P*<0.001, **** *P*<0.0001.

To understand the physiological changes behind the increase in gut microbiota of infected mosquitoes, we analyzed the expression of known gut-homeostasis-related genes. One such gene is the gut-membrane-associated protein (*MESH*), which recently has been recognized to be part of the insect gut homeostatic mechanism [37]. Our analysis showed no change in *MESH* expression in the midgut at 3d PI but a significant increase in the midgut of *B*. *bassiana* and *B*. *brongniartii*-infected mosquitoes at 6d PI (Fig 6A). These coincided with the increases of bacterial load observed in our microbiome load assessment (Fig 5B). Upregulation of *MESH* expression due to an increase in midgut microbiota has been reported previously for the *Ae*. *aegypti* mosquito [37]. Given that MESH is known to control the expression of *DUOX*, we next decided to evaluate *DUOX1* and *DUOX2* expression in the midgut of infected mosquitoes. We observed no changes in the expression of *DUOX1* at 3d PI, but a substantial increase in expression at 6d PI in the gut of *B*. *bassiana* and *B*. *brongniartii*-infected mosquitoes (Fig 6B). No significant regulation of *DUOX1* was observed in the midguts of *T*. *roseum* or *I*. *javanica* at 3d or 6d PI. In addition, no change in the *DUOX2* expression profiles were observed in the midgut of mosquitoes from any of the treatments. Since *DUOX1* is directly related to ROS production in the midgut we assessed ROS activity levels by measuring hydrogen peroxide (H_2_O_2_) and superoxide production by the midgut. Our assessment shows a significant decrease of H_2_O_2_ release (Fig 6C) as well as a decrease in superoxide staining by the midgut epithelium (Fig 6D) at 6d PI. This decrease was independent of whether the mosquito was challenged with an entomopathogenic or non-entomopathogenic fungal isolate, since *T*. *roseum*-infected mosquito midguts released 30% less H_2_O_2_ than controls, compared to the 20% reduction seen in mosquitoes exposed to *B*. *bassiana* or *B*. *brongniartii* or 24% in mosquitoes challenged with *I*. *javanica* (Fig 6C). These results were consistently observed in three independent experiments. Next, we assayed the expression of antioxidant genes that would counter act any effects from ROS levels. No significant changes were observed in the midgut expression of *CuSOD2*, *GPX*, and Catalase gene (*CAT*) at 3d or 6d PI, while a significant *TPX* up-regulation at 6d PI was observed only in *B*. *brongniartii*-infected mosquito midguts (Fig 6E). Overall, fat body tissues had a more robust modulation of *DUOX* genes as well as antioxidant genes especially at 6d PI with a significant *DUOX2* upregulation in the fat body of *B*. *brongniartii*-infected mosquitoes (Fig 6F and S4 Fig). Of the four different antioxidant genes evaluated, *CuSOD2* and *TPX* were the only two genes whose expression was significantly upregulated in either *B*. *bassiana* or *B*. *brongniartii*-infected mosquito fat bodies (Fig 6F and S4 Fig). There was no significant regulation of any of these genes in fat bodies of *I*. *javanica* or *T*. *roseum*-infected mosquitoes.

To assess whether the gut bacteria had any influence in the mosquito survival to fungal infection, mosquitoes were treated with antibiotics from the day prior to infection until 15d PI. The antibiotic treatment was successful in clearing the mosquito gut bacteria (S5 Fig), but it did not change the survival rate of the fungal-infected mosquitoes (S6 Fig). This was observed even when mosquitoes were exposed to a higher concentration of fungal conidia (S7 Fig).

## Discussion

Our comparison study with fungal strains of diverse virulence shows a dynamic interaction of immune signaling pathways and effectors working in concert to control the infection. The observation of blastospores in mosquito hemolymph coupled with the presence of mycelial growth in mosquito cadavers confirmed the successful infection by these entomopathogenic strains. In comparison, the absence of blastospores but presence of mycelial growth on mosquito cadavers from the *T*. *roseum*-challenged group indicated the true nature of this fungus, since saprophytic fungi are known to grow on dead organic matter, including dead insects.

Upon immune challenge, pathogen recognition is the first critical step in the elicitation of immune responses mounted to control pathogen invasion. The fungal recognition protein *CLSP2* and the anti-fungal effector *Tep22* have been recently identified as part of the mosquito anti-fungal response [11]. In a series of experiments Wang *et al* [11] described elevated levels of expression for these two molecules at 24h post-infection with *B*. *bassiana*. Our results corroborate the elicitation of *CLSP2* and *TEP22* and show increasing fat body expression of these two genes at the later times of infection, 3d and 6d PI. Fungal recognition by these two molecules was independent of the fungal strain and the magnitude of their regulation appears to reflect the virulence of the infecting fungi, with *B*. *bassiana*-infected mosquitoes showing the strongest *CLSP2* upregulation at 3d and 6d PI. This indicates that the degree of immune elicitation is fungal strain-dependent.

The strong expression of the anti-fungal effector *TEP22* in the fat body of infected mosquitoes at 3d PI indicates the attempt by the mosquito’s immune system to control the infection. It is interesting to note that while *TEP22* expression increases with time, as blastospores start to disseminate throughout the mosquito body, its negative regulator *CLSP2* also increases in expression. This suggests a tight control of the response as the infection progresses. Furthermore, while *B*. *bassiana* is eliciting the strongest expression of this antifungal effector, mosquitoes are nevertheless unable to limit the infection and succumb two to five days earlier than mosquitoes exposed to *B*. *brongniartii* or *I*. *javanica*. This could mean that while *TEP22* is somewhat effective at limiting *I*. *javanica* and *B*. *brongniartii* proliferation and dissemination; it is less so with regards to *B*. *bassiana*.

Pathogen recognition leads to the elicitation of immune signaling pathways that would direct anti-microbial immune responses. Our study shows a degree of compartmentalization for each of the immune signaling pathways in response to fungal infection. While the transcription factor *REL1* (Toll pathway) is elicited in the fat body especially at the later stages of infection, it remained unchanged in the midgut compartment. This is in contrast to *REL2* expression (IMD pathway), which largely showed no significant expression at 3d PI, but displayed a robust expression in the midgut and fat body of infected mosquitoes at 6d PI. These results are in line with what has been reported previously in *Drosophila* in that the Toll pathway is mostly expressed in hemocytes and fat body for cellular and humoral immunity [42], while IMD pathway acts both at the midgut and fat body for epithelial and humoral immunity [43–45]. Whereas the Toll pathway is known to be involved in the antifungal defense in both fruit flies [46] and mosquitoes [16, 47], the IMD pathway is generally regarded as part of the antibacterial and anti-plasmodial defense [16, 48]. Interestingly, our results indicate that the IMD pathway may be playing a bigger role in the anti-fungal defense, since its transcription factor was highly elicited with all three entomopathogenic fungi. Alternatively, the IMD pathway activation in the midgut might occur in response to the increase in the microbial gut flora observed at the later stages of infection, controlling or preventing a systemic bacterial infection. Another interesting observation was that, with exception to the activation of the Toll pathway at 3d PI in the fat body, the IMD pathway was the only immune signaling pathway elicited in *I*. *javanica*-infected mosquitoes. This suggests that while the mode of invasion is relatively similar among all entomopathogenic fungi, its dissemination and development as blastospores inside the hemocoel might interact differently with the mosquito immune system.

In addition, the expression of *STAT* and *JNK*, from the JAK-STAT and JNK pathways respectively, showed additional modulation of pathway activation in relation to the invading fungal strain. The strong elicitation of the *STAT* pathway in *B*. *bassiana* and *B*. *brongniartii*-infected mosquitoes, but not in *I*. *javanica*, is likely the result of specific fungal-mosquito interactions. For instance, it could be in response to fungal secondary metabolites modulating the mosquito immune response. Prior research has found that JAK-STAT pathway is crucial in the anti-fungal defense against *B*. *bassiana* [47]. Although further analyses are needed, our results might indicate a potential divergence in this response, given the lack of elicitation of this pathway in *I*. *javanica*-infected mosquitoes. Although less is known about the importance of the JNK pathway in the anti-fungal response, the fact that this pathway was strictly elicited in *B*. *brongniartii*-infected mosquitoes, reflect specific interactions that occur between each fungal strain and the mosquito. Pathogen-specific elicitation of immune signaling pathways is not uncommon and has been observed in the *Drosophila*-bacteria [43, 49] and in the *Anopheles*-*Plasmodium* system [50]. Hence, a level of pathogen-specific modulation of immune signaling pathways is likely to occur with different fungal strains.

One important picture that emerged in our expression analysis is that the main signaling pathways appear to be elicited consistently at the later stages of infection, while largely remaining unchanged relative to the controls at the early 3d PI time point. This could be the result of early active-immune suppression by the entomopathogenic strains or the result of immune evasion by fungal morphological changes (blastospores). Indeed, prior studies on entomopathogenic fungi metabolism have described the existence of bioactive factors with potent immune suppressive activity [9, 51]. *B*. *bassiana* as well as *B*. *brongniartii* are known to produce several of these compounds [52]. Whether any of these bioactive compounds are directly affecting these immune signaling pathways in the mosquito remains to be investigated. Thus, while immune suppression is possible, the lack of immune signaling activation might be due to the lack of sufficient recognition of blastospores inside the mosquito body [53]. This would agree with what is currently known about blastospores in that they are devoid of most of the structural cell wall found in conidia or mycelia that would elicit the immune system and hence, go largely unnoticed by hemocytes [9]. In addition, the weak and irregular modulation of three of the four antimicrobial peptides tested may suggest a level of immune suppression by the fungal entomopathogen. For instance, *CECG* and *DEFC* were only significantly expressed at 6d PI in *B*. *bassiana*-infected mosquitoes but presented an irregular and not significant expression in mosquitoes infected with the other two fungal entomopathogens. Alternatively, it may indicate that the expression of these effectors is not directly modulated by the fungi but it is an indirect effect of fungal infection, for instance, they could be elicited in response to the increase in gut microbiota. The only antimicrobial peptide consistently upregulated across all the different entomopathogenic fungal infections was *LYSC*. The significant regulation of this AMP in the fat body at 3d and 6d PI might suggest that it is playing a bigger role in the antifungal defense compared to the other two AMP genes tested.

Another important immune response mounted against invading fungal pathogens is the melanization cascade [21, 54, 55]. Although phenoloxidase activity has been attributed primarily to hemocytes [56], it has also been localized in tissues such as the hindgut [57], trachea, and adult cuticle [58]. Detailed analysis during the acute phase (24h) of *B*. *bassiana* infection has shown the elicitation of several prophenoloxidase genes [11]. Our analysis at 3d and 6d PI revealed the downregulation of two important members of the melanization cascade (*PPO3* and *PPO5*). In addition, we observed a significant reduction in phenoloxidase activity in the whole body of *B*. *bassiana* and *B*. *brongniartii*-infected mosquitoes at these same time points of infection. These indicates active modulation of this antifungal defense mechanism. While the reduction in PO activity could be due to active immune suppression by the entomopathogenic fungi, it is plausible that it is regulated by the mosquito itself. In fact, *CLSP2* has been found to negative modulate the transcription of PPO genes during fungal-infection [11]. Although, Wang et al [11] did not report downregulation of PPO genes, silencing *CLSP2* led to an increase in the expression of these same phenoloxidase genes at 24h PI. In our studies, significant *CLSP2* upregulation coincided with significantly lower *PPO3* and *PPO5* expression at the later stages of *B*. *bassiana* and *B*. *brongniartii* infection. A similar reduction in PO activity at the later times of infection has been observed following infection with *B*. *bassiana* in the moth *Spodoptera litura*, the fruit fly *Drosophila melanogaster* and the wax worm *Galleria mellonella* [55, 59, 60]. Another line of evidence for lower PO activity resides in the upregulation of lysozyme and *REL2* expression post-fungal infection. Our analysis indicates a significant increase in lysozyme expression in the fat body at 3d and 6d PI. Lysozyme has been found to negatively regulate PPO activation upon an immune challenge [61, 62]. In addition, *REL2* has been shown to negatively regulate melanization in the mosquito *Anopheles gambiae* [18]. Our expression analysis showed a significant increase in *REL2* expression at the later stages of infection when PO activity was the lowest particularly for *B*. *bassiana* and *B*. *brongniartii*-infected mosquitoes. Thus, this potential tradeoff between PO activity and lysozyme and/or *REL2* regulation could be a mosquito strategy to avoid the burst of the most costly and toxic PO activation. Alternatively, the drop in PO activity, especially at the later time points, could be the result of an exhaustion of the melanization pathway components, as they are being used to counteract the continuous proliferation of fungal bodies. However, a study by Matskevich et al. [59], who also observed suppression of PO activity, found similar amounts of prophenoloxidase proteins in naive and *B*. *bassiana*-infected *D*. *melanogaster* fruit flies. Hence, whether this reduction in PO activity is the result of immune suppression by the entomopathogenic fungi or a protective process developed by the mosquito and exploited by the entomopathogenic fungi, remains to be elucidated.

The infection-modulated expression of some immune genes led us to investigate the microbial flora of infected mosquitoes since their elicitation might indirectly affect the midgut microbiome. To our surprise we observed an increase in the midgut bacterial load of infected mosquitoes at the later stages of fungal infection. The level of midgut microbiota increase appears to reflect the magnitude of the immune response, since mosquitoes that had the most immune elicitation (*B*. *bassiana* and *B*. *brongniartii*-infected mosquitoes) also had the highest increase in gut microbiota. Hence, this increase in midgut bacterial load is most likely the indirect result of the multifaceted response mounted by the mosquito against the fungal challenge. A microbiome analysis across all treatment groups showed no large differences on the commensal gut bacteria composition. Thus, the bacterial load increase in the midgut of infected mosquitoes likely occurs independent of bacteria taxa. A recent article by Wei et al. [63] also describes an increase in gut bacteria loads following *B*. *bassiana* infection in *Anopheles stephensi* mosquitoes. Furthermore, the authors found that the increase in bacteria accelerated the death of infected mosquitoes. Our comparative study using antibiotic-treated and untreated mosquitoes did not show any significant difference with regards to survival. The difference between the results described by Wei et al. and our work could be attributed to the use of different mosquito species and different fungal strains, or due to differences in bacterial composition in the gut of mosquitoes. Our study evaluated the commensal midgut bacteria during the course of an entomopathogenic fungal infection, and it could be expected that pathogenic bacteria would produce potentially different phenotypes. Although our results show no detrimental effect of the microbial load increase in the survival of infected mosquitoes, it could still alter vector competence. This could be a potential phenotype given that the midgut microbiota has been shown to influence arboviral and parasitic infection of the mosquito midgut [64–66].

To decipher any mechanisms that were leading to the increase in midgut microbial load, we evaluated the expression of genes involved in maintaining gut homeostasis [37, 49]. The dual oxidase (Duox)-generation of reactive oxygen species (ROS) has been identified to be a critical mechanism controlling the proliferation of gut bacteria. This mechanism, in turn, has been shown to be under the control of MESH, a gut-membrane-associated protein [37]. In addition, the Mesh-mediated *DUOX* induction is known to be elicited in response to bacteria proliferation in the gut. Hence, *MESH* upregulation in the gut of *B*. *bassiana* and *B*. *brongniartii*-infected mosquitoes at 6d PI is likely a secondary response to the increase in gut bacteria, especially since *MESH-DUOX* expression were absent at the early time of 3d PI.

The significant reduction in soluble H_2_O_2_ (a potent ROS) being released by the midgut of fungal-challenged mosquitoes most likely explains the increase in bacterial loads. In addition, the gene expression analysis of the Duox pathway and other detoxifying enzymes suggests that there is a post-translational regulation of the DUOX enzyme. Studies have found that peptidoglycan from commensal bacteria did not induce DUOX enzyme activity even though it did induce *DUOX* gene expression [67, 68]. This is a controlled regulation that prevents overstimulation and generation of potentially deleterious ROS in the absence of pathogenic gut bacteria [68]. Alternatively, it could also be part of the antifungal immune response profile, directing and localizing ROS production where the fungal pathogen is detected (hemocoel) and not in the gut, where commensal bacteria are located. Further studies are needed to discern the significance of this phenotype.

In comparison to the midgut, the fat body showed high elicitation of both oxidant-generating and detoxifying enzymes in mosquitoes infected with the entomopathogenic fungal strains. This expression profile most closely represents the systemic immune response that might be expected at the later stages of infection. Interestingly, *T*. *roseum*-challenged mosquitoes also had a significant drop in H_2_O_2_ release in three independent experiments. The limited transcriptome analysis conducted in this study does not allow us to determine a possible mechanism for this phenotype in *T*. *roseum*, but it might indicate that this is part of the antifungal response when challenged with fungal spores, independent of whether they are pathogenic or not. Further studies are needed to determine the implications of this response in *T*. *roseum*.

In summary, our study shows that the antifungal immune response of the mosquito *Ae*. *aegypti* displays a degree of compartmentalization that changes as the fungal entomopathogen disseminates throughout the body of the mosquito during the infection process. Our results further show a tissue-specific immune modulation that in turn varies according to the fungal entomopathogen infecting the mosquito. The concomitant differential elicitation of immune pathways along with the modulation of redox signaling pathways appear in turn to alter midgut homeostasis. This indicates that fungal infection have far reaching effects to other microbes that naturally reside in mosquitoes, which could in turn alter the vector competence of infected mosquitoes.

Our study demonstrates an intricate mosquito-fungi interaction, which, despite fungal recognition and immune elicitation by the mosquito, results in the death of the host. This observation, in turn, indicates that the fungi may play a more complex role in suppressing or modulating mosquito immunity than has been previously recognized. In addition, this study contributes to a better understanding of mosquito-fungi interactions that may facilitate the development of novel fungal-based biocontrol strategies, for instance, through the selection of entomopathogenic fungi with different modes of action.

## Supporting information

S1 Fig Phylogenetic relationships of the fungal isolates used in this study (in red). Tree constructed using the maximum Likelihood method based on a General Time Reversible model. The bootstrap support was calculated from 1000 replicates and it is shown over the branches.
(TIF)

S2 Fig Antimicrobial peptide gene expression in the midgut and fat body tissues at 3d and 6d PI. Data represents the fold change in expression from at least three independent experiments. Data was log2-transformed and analyzed by one-way ANOVA with Dunnett’s post-test. * *P*<0.05, ** *P*<0.01, *** *P*<0.001, **** *P*<0.0001.
(TIF)

S3 Fig Mosquito midgut bacterial diversity following fungal-infection. **(A)** Bacterial composition at the family level in control (Ct), *B*. *bassiana* (Bb), *T*. *roseum* (Tr), *B*. *brongniartii* (Br) and *I*. *javanica* (Ij)-infected groups. **(B)** Bacterial diversity measurements based on OTUs (97%) from each of the treatment groups. Data was analyzed by one-way ANOVA with Dunnett’s post-test.
(TIF)

S4 Fig Oxidant and antioxidant enzyme gene expression in the midgut and fat body tissues at 3d and 6d PI. Data represents the fold change in expression from at least three independent experiments. Data was log2-transformed and analyzed by one-way ANOVA with Dunnett’s post- test. * *P*<0.05, ** *P*<0.01, *** *P*<0.001, **** *P*<0.0001.
(TIF)

S5 Fig Bacterial growth from midgut macerates of control and antibiotic-treated mosquitoes. (TIF)

S6 Fig Kaplan-Meier survival curves of antibiotic-treated and control mosquitoes under the context of fungal infection. **(A)** *B*. *bassiana*, **(B)** *I*. *javanica*, **(C)** *B*. *brongniartii* and **(D)** *T*. *roseum*.
(TIF)

S7 Fig Kaplan-Meier survival curves of antibiotic-treated and control mosquitoes under the context of a fungal infection with a higher dose. **(A)** *B*. *bassiana*, **(B)** *I*. *javanica*, **(C)** *B*. *brongniartii* and **(D)** *T*. *roseum*.
(TIF)

S1 Table Primer sequences used in qPCR. (XLS)

S2 Table Calculated LT_50_ and LT_95_ values for each of the fungal isolates used against adult *Aedes aegypti*. ND (not determined) indicates that values could not be calculated because mortality was lower than 50% or 95% at the end of the experiment.
(XLSX)
