# Supplementary figures and images for: Entomopathogenic fungal infection leads to temporospatial modulation of the mosquito immune system
Source: PLoS Negl Trop Dis. 2018 Apr 23;12(4):e0006433. doi: 10.1371/journal.pntd.0006433 (PMC5933799; doi:10.1371/journal.pntd.0006433)

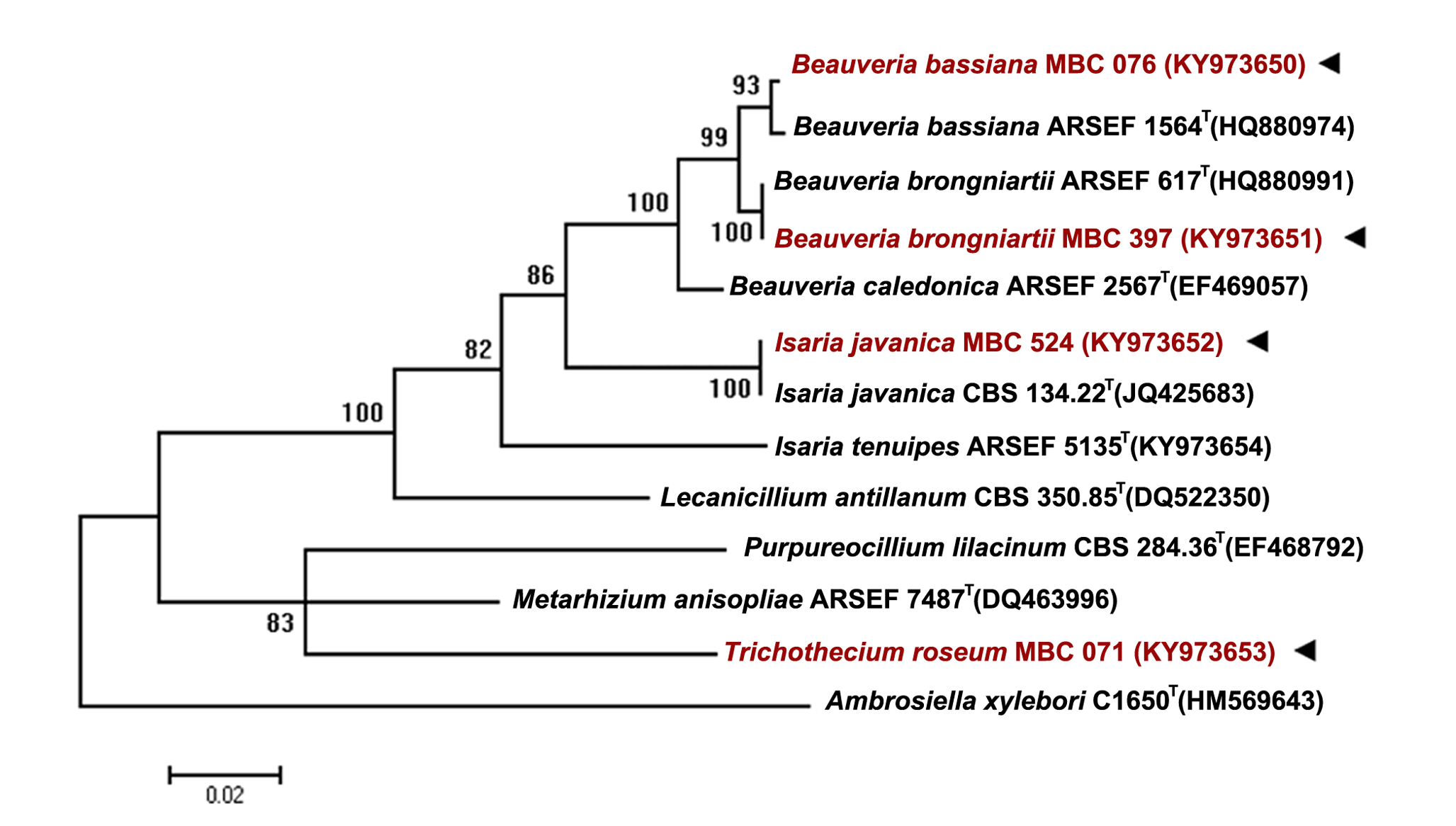

Supplement: S1 Fig — Tree constructed using the maximum Likelihood method based on a General Time Reversible model. The bootstrap support was calculated from 1000 replicates and it is shown over the branches. (TIF) [file pntd.0006433.s001.tif]

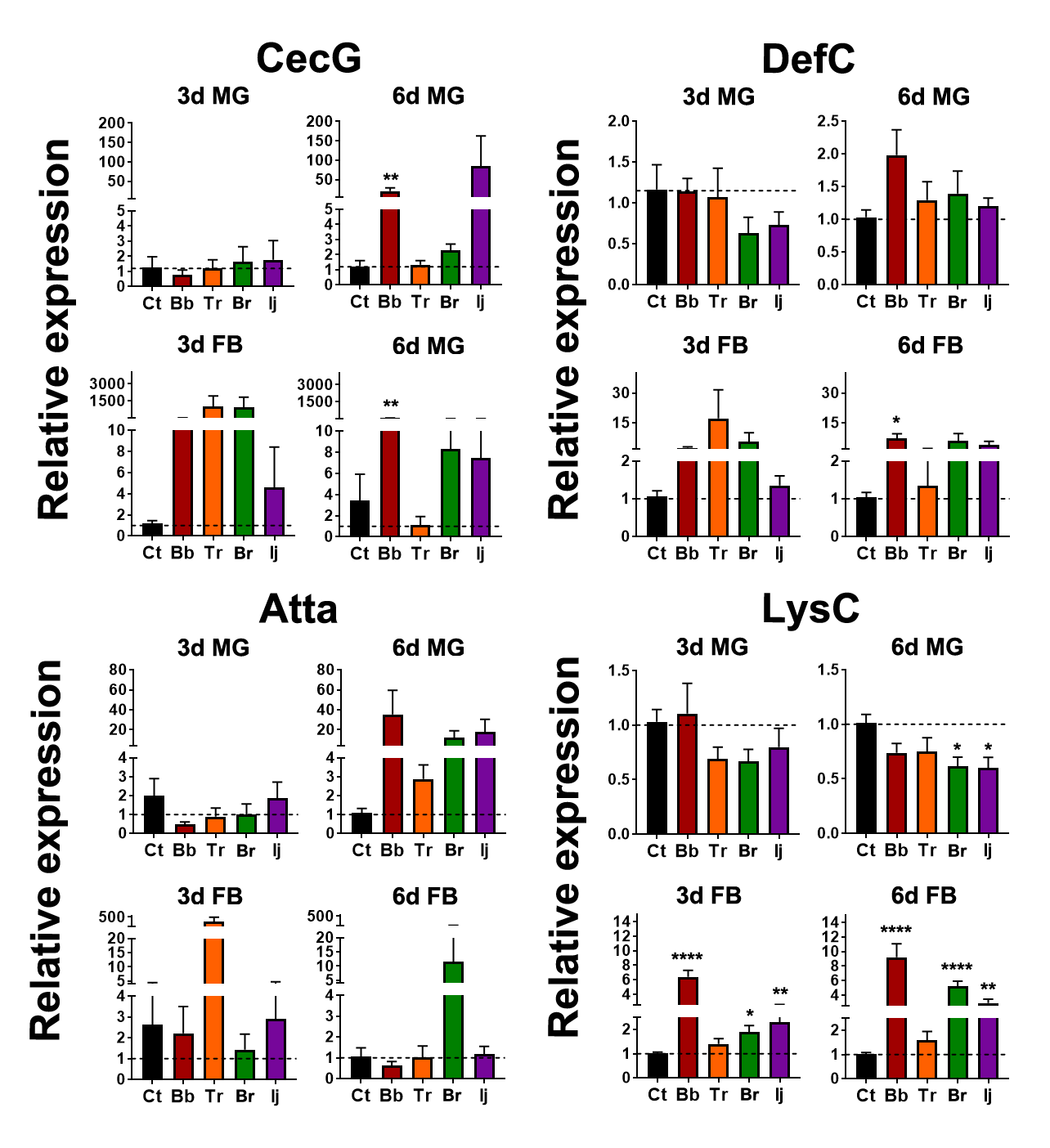

Supplement: S2 Fig — Data represents the fold change in expression from at least three independent experiments. Data was log2-transformed and analyzed by one-way ANOVA with Dunnett’s post-test. * P<0.05, ** P<0.01, *** P<0.001, **** P<0.0001. (TIF) [file pntd.0006433.s002.tif]

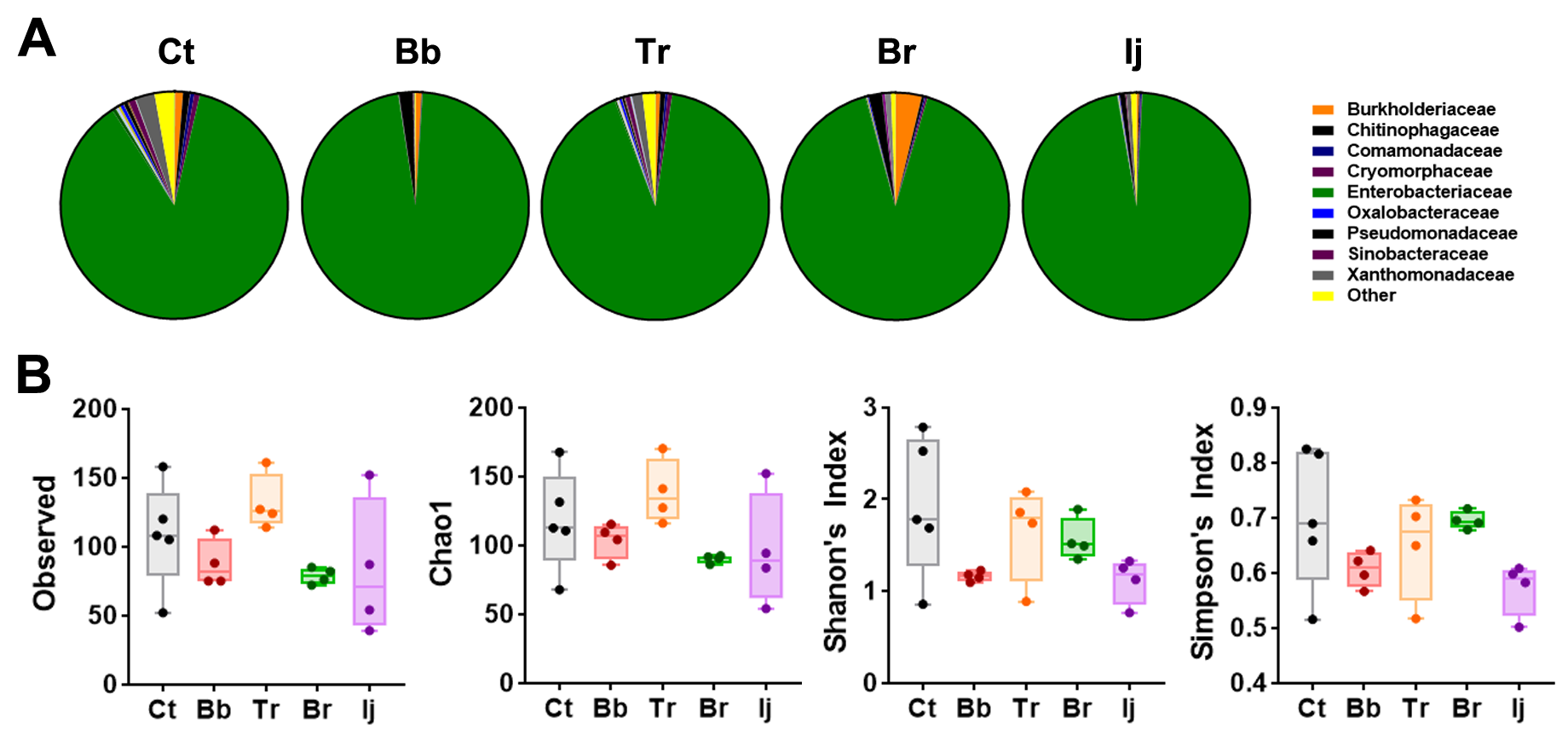

Supplement: S3 Fig — (A) Bacterial composition at the family level in control (Ct), B. bassiana (Bb), T. roseum (Tr), B. brongniartii (Br) and I. javanica (Ij)-infected groups. (B) Bacterial diversity measurements based on OTUs (97%) from each of the treatment groups. Data was analyzed by one-way ANOVA with Dunnett’s post-test. (TIF) [file pntd.0006433.s003.tif]

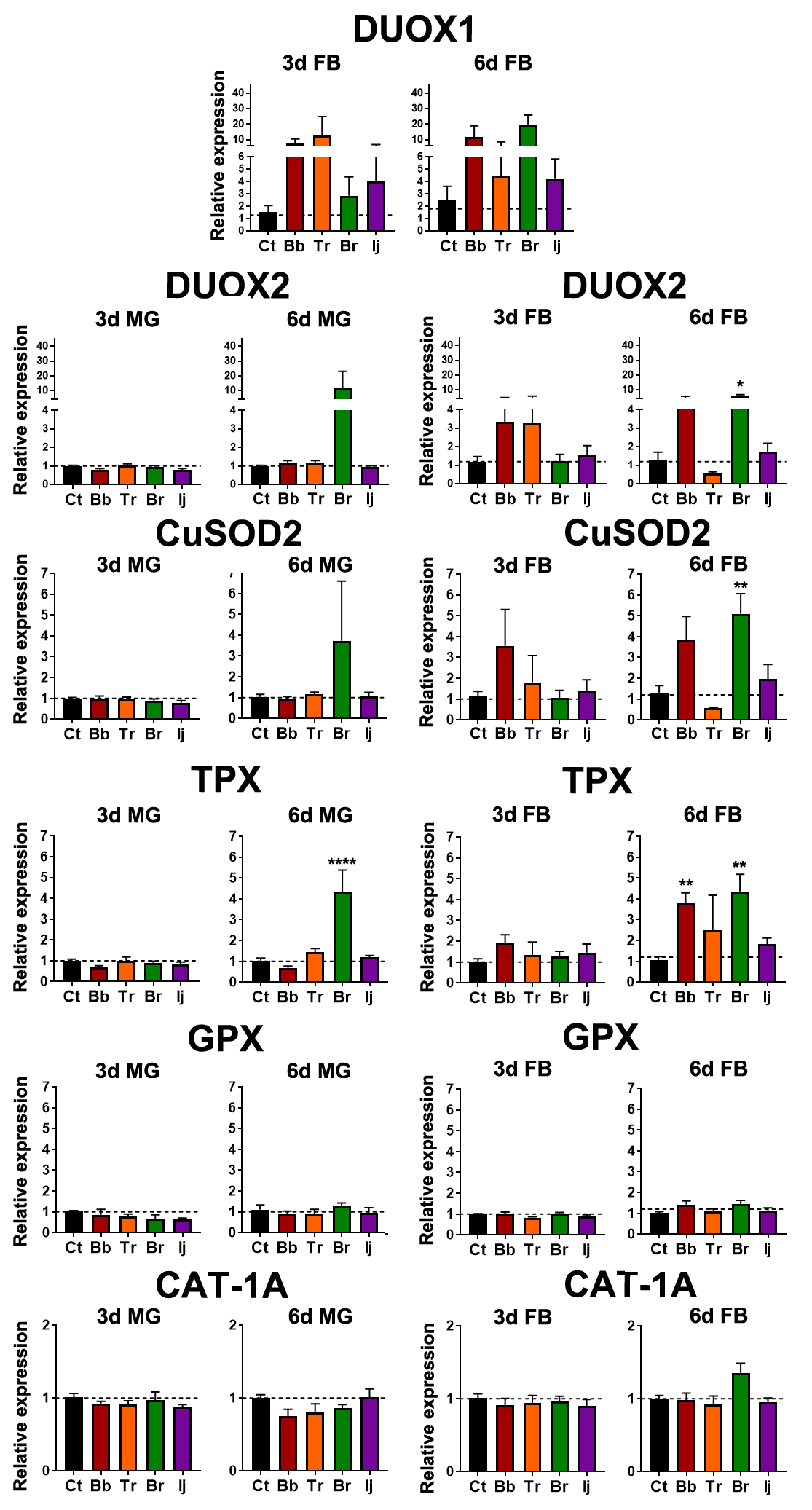

Supplement: S4 Fig — Data represents the fold change in expression from at least three independent experiments. Data was log2-transformed and analyzed by one-way ANOVA with Dunnett’s post- test. * P<0.05, ** P<0.01, *** P<0.001, **** P<0.0001. (TIF) [file pntd.0006433.s004.tif]

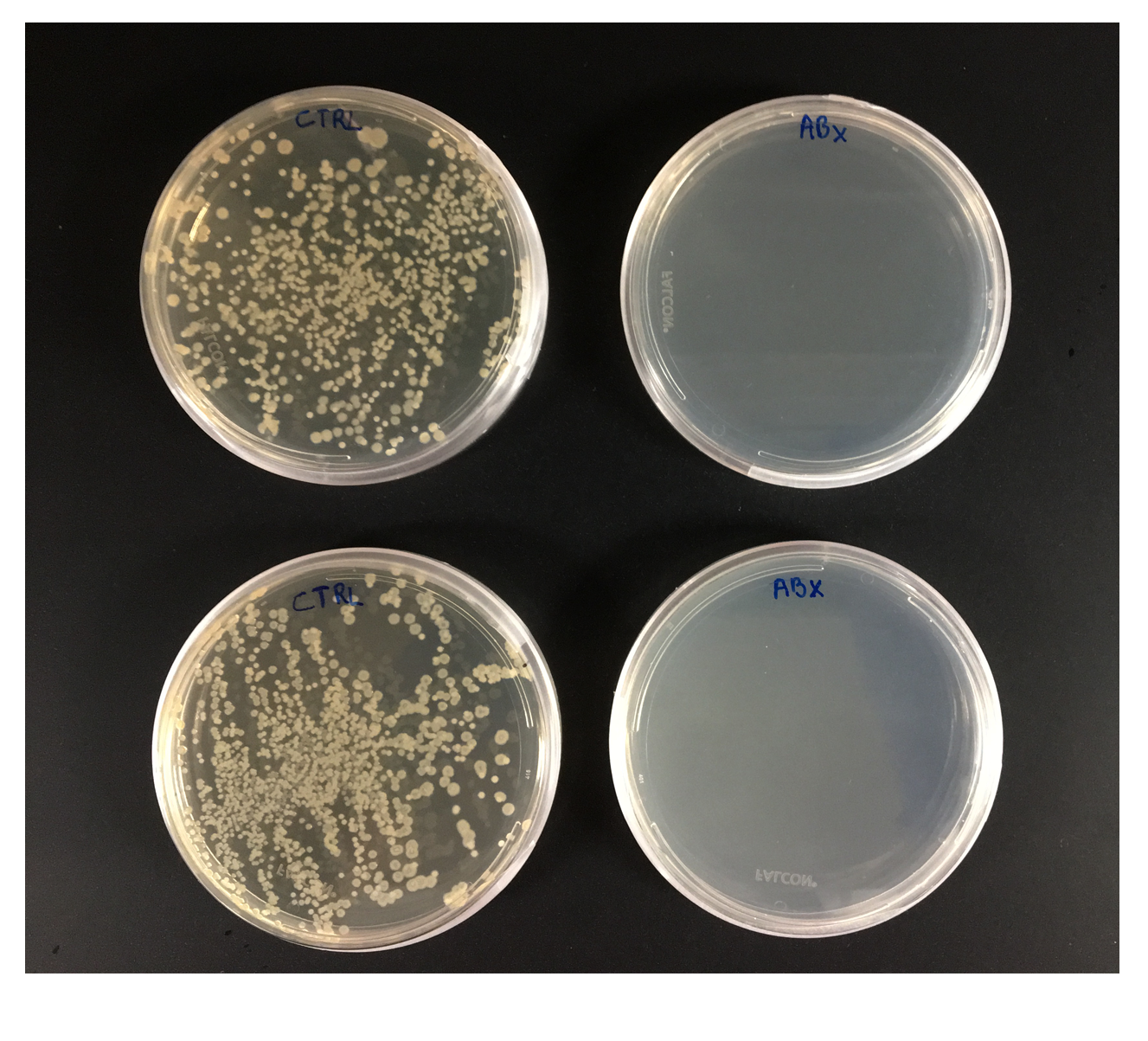

Supplement: S5 Fig — (TIF) [file pntd.0006433.s005.tif]

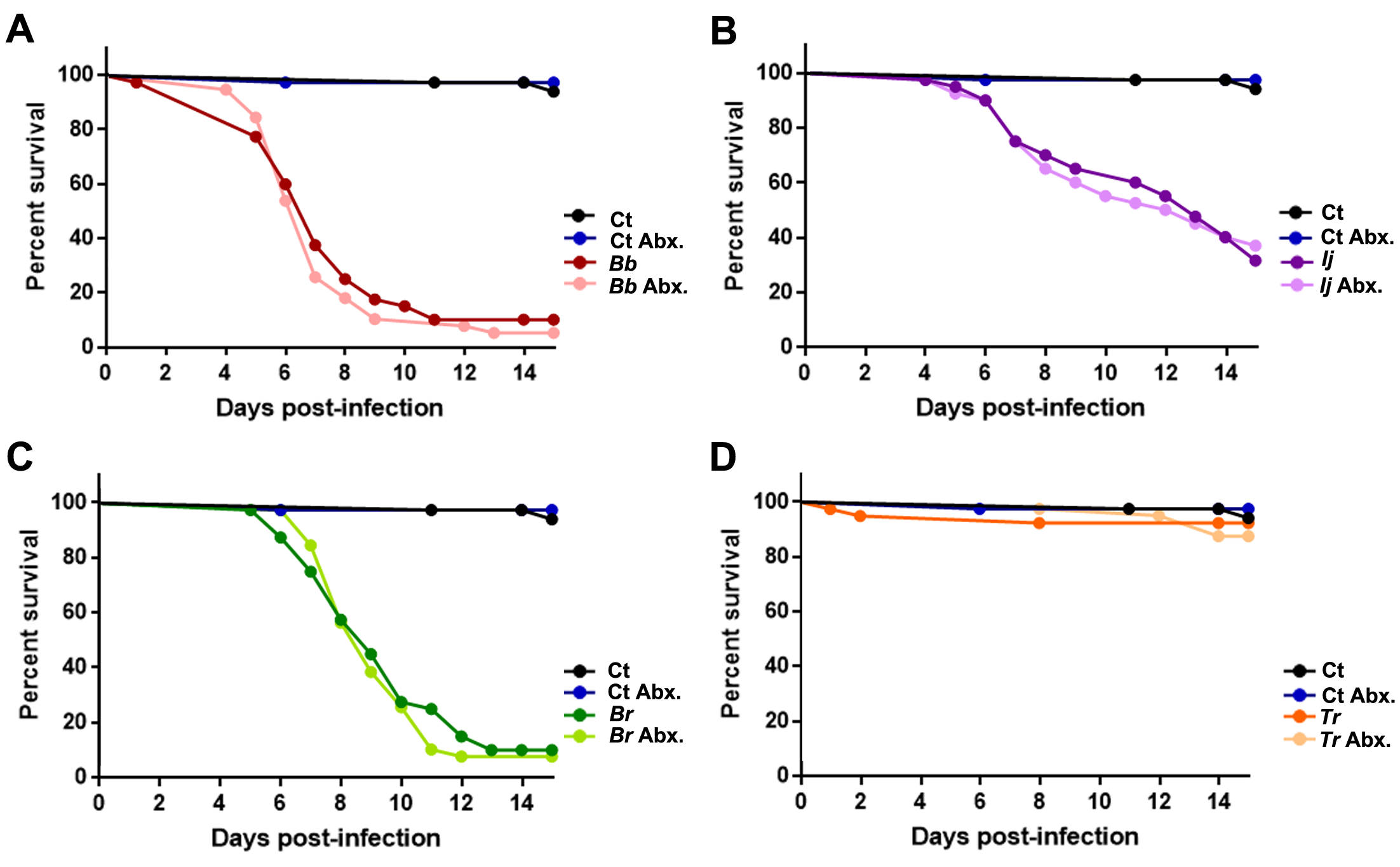

Supplement: S6 Fig — (A) B. bassiana, (B) I. javanica, (C) B. brongniartii and (D) T. roseum. (TIF) [file pntd.0006433.s006.tif]

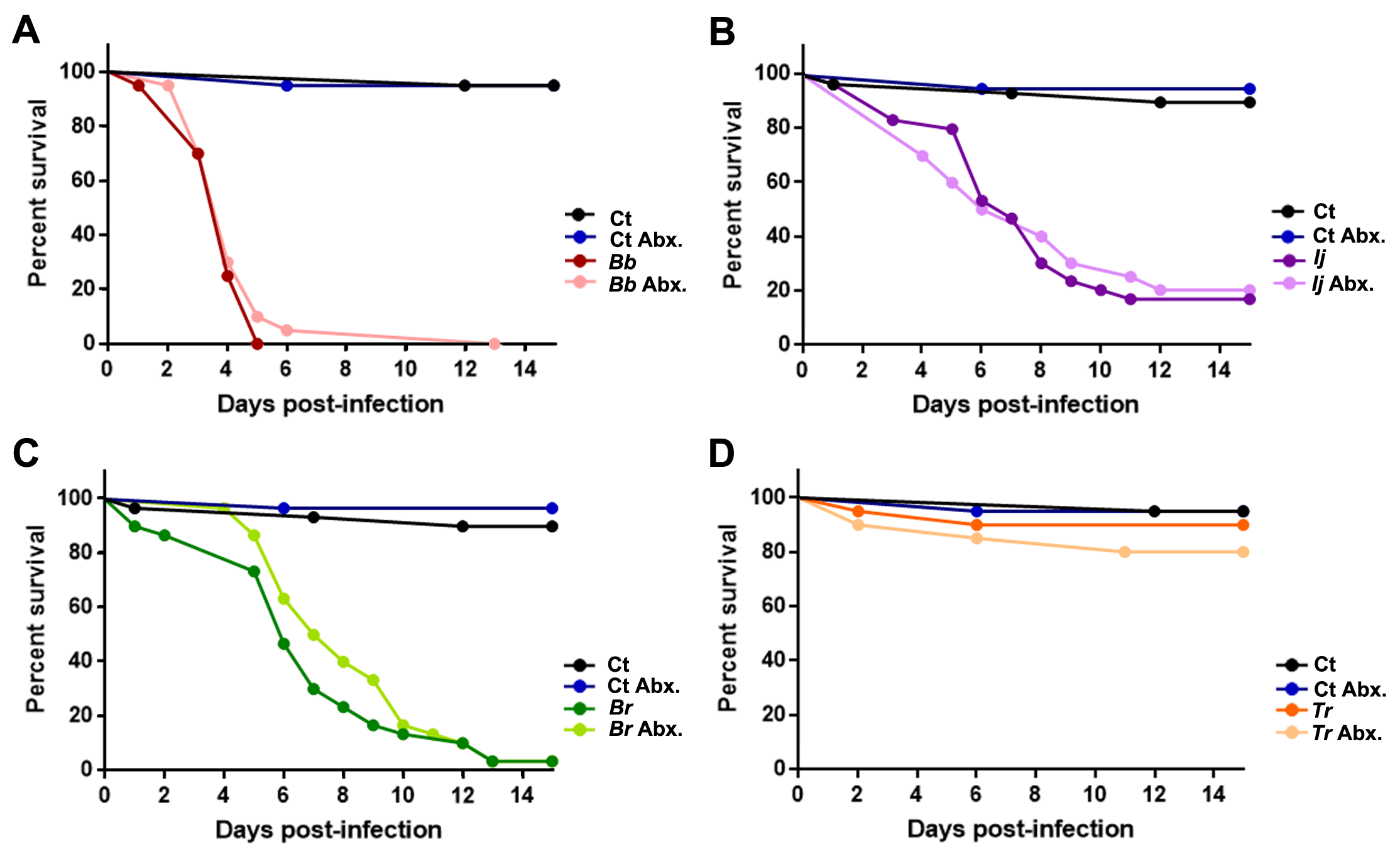

Supplement: S7 Fig — (A) B. bassiana, (B) I. javanica, (C) B. brongniartii and (D) T. roseum. (TIF) [file pntd.0006433.s007.tif]
